# The RNA‐Binding Protein PARN Remodeled 3′ UTR Structure Defines Poly(A)‐Loading Sites to Mediate Immunoglobulin Homeostasis

**DOI:** 10.1002/advs.75609

**Published:** 2026-05-12

**Authors:** Siyuan Sun, Chen Yang, Xiaoyu Wang, Naijing Hu, Shengyao Zhang, Xinchun Li, Yu Chen, Jianan Zhai, Xiangfeng Tang, Wei Lu, Chenxu Lu, Changchang Cao, Weiru Yu, Wenhao Wu, Xiaonan Nie, Fengchao Wang, Bing Fang, Fazheng Ren, Yixuan Li, Juan Chen

**Affiliations:** ^1^ Key Laboratory of Precision Nutrition and Food Quality Department of Nutrition and Health China Agricultural University Beijing China; ^2^ State Key Laboratory of Ophthalmology Optometry and Vision Science Eye Hospital Wenzhou Medical University Wenzhou China; ^3^ College of Food Science and Nutritional Engineering China Agricultural University Beijing China; ^4^ Department of Clinical Laboratory Peking University People′s Hospital Beijing China; ^5^ National Engineering Laboratory for Birth Defects Prevention and Control of Key Technology Beijing Key Laboratory of Pediatric Organ Failure Department of Pediatrics the Seventh Medical Center of Chinese PLA General Hospital Beijing China; ^6^ State Key Laboratory of Cardiovascular Disease National Center for Cardiovascular Diseases Fuwai Hospital Chinese Academy of Medical Sciences and Peking Union Medical College Beijing China; ^7^ National Institute of Biological Sciences Beijing China

**Keywords:** alternative polyadenylation (APA), class switch recombination (CSR), poly(A)‐specific ribonuclease (PARN), RNA binding protein (RBP), systemic lupus erythematosus (SLE)

## Abstract

Class Switch Recombination (CSR) is essential for generating high‐affinity antibody isotypes from IgM during adaptive humoral responses. Despite well‐established roles for various transcription factors, whether CSR is subject to dedicated post‐transcriptional control represents a significant gap in knowledge. By integrating conditional knockout models with SLE disease contexts, this study is the first to identify that the RNA‐binding protein, poly(A)‐specific ribonuclease (PARN), serves as a key positive regulator of antibody‐secreting cell function. Mechanistically, PARN preferentially binds 3′ UTRs and enhances the utilization of proximal poly(A) sites on a genome‐wide scale in vivo. Further results show that PARN binds UGUA and AA(U/A)AAA upstream elements to form a specific spatial RNA–protein complex, through which it exerts exonuclease activity to shorten poly(A) tails, thereby decreasing mRNA stability. In addition, we identified a class of functional genes—including *Foxp1*—whose dynamic 3′ UTR changes directly regulate antibody secretion. This study reports a novel post‐transcriptional mechanism by which PARN promotes antibody production through modulation of 3′ UTR length. These findings not only advance our understanding of humoral immune regulation but also highlight a potential therapeutic target for autoimmune diseases such as SLE.

## Abstract 2

In antibody‐secreting cells, PARN recruits PTBP1 to bind UGUA/AAUAAA motifs, globally modulating 3′ UTR length via alternative polyadenylation. PARN promotes the short, less stable *Foxp1* isoform, reducing *Foxp1* expression and facilitating class switch recombination. PARN deficiency elevates *Foxp1*, suppressing CSR. Conversely, PARN overexpression in SLE drives excessive antibody production by lowering *Foxp1*.

## Graphical abstract

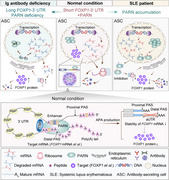

## 1 Introduction

B cells produce a diverse repertoire of antibodies that mediate immune clearance of tumors [1], viruses [2], and various microorganisms [3]. Within germinal centers, B cells undergo class switch recombination (CSR), during which recombination occurs between the Cµ constant exon and one of the downstream constant exons (*C*γ, *C*α, or *C*ε), leading to the production of high‐affinity IgG, IgA, or IgE antibodies [4]. Dysregulation of CSR is implicated in diseases such as Hyper IgM Syndromes [5] (HIGM) and systemic lupus erythematosus [6] (SLE). During antibody production, the immunoglobulin heavy chain (IgH) locus undergoes precisely regulated transcription, generating large amounts of RNA [7]. However, the metabolism, processing, and modification of these abundant transcripts in such processes are not fully understood. Alternative polyadenylation (APA), as a crucial regulatory mechanism for maintaining cellular function, has also emerged as a potential biomarker for disease diagnosis and therapy [8]. Nevertheless, how APA influences antibody transcript levels and isotype selection has not been determined.

RNA‐binding proteins (RBPs) represent a class of proteins that interact with diverse RNA molecules within cells [9]. By participating in RNA capping, splicing, polyadenylation, stabilization, and localization, RBPs act as central regulators of post‐transcriptional gene expression [10, 11]. In the immune system, growing evidence has revealed pivotal roles of RBPs in regulating B cell activation, antibody diversification, and CSR [12, 13, 14]. For instance, ROD1 collaborates with the cytidine deaminase enzyme AID to facilitate CSR initiation by binding to ssDNA [15]. The RNA helicase DDX1, recognizes G‐quadruplex (G4) structures within intronic switch transcripts and remodels them into S‐region R‐loops, thereby recruiting AID to specific genomic loci [16]. HNRNPU stabilizes the non‐homologous end joining (NHEJ) ribonucleoprotein complex and prevents aberrant DNA repair by promoting proper R‐loop accumulation [17]. Additionally, HuR controls cell proliferation and DNA damage pathways in germinal center B cells, acting as a vital RNA splicing modulator for high‐affinity antibody production [18]. Collectively, these findings underscore the active role of RBPs in shaping antibody production and immune homeostasis. However, the global post‐transcriptional landscape of RBP involvement in CSR remains poorly defined, and the mechanisms through which RBPs coordinate APA events are still largely unexplored.

In eukaryotic cells, balancing mRNA synthesis and degradation is essential for maintaining transcriptome homeostasis. Deadenylation, the progressive removal of adenosine residues from the poly(A) tail, serves as the initial and rate‐limiting step in mRNA decay among the processes that regulate mRNA turnover [19]. Poly(A)‐specific ribonuclease (PARN) is a conserved 3′–5′ mammalian exonuclease that preferentially binds the poly(A) tail and interacts with the 5′ cap structure in vitro [20]. Although PARN has been identified as a major deadenylase responsible for shortening extended poly(A) tails [21, 22] and has been linked to several human diseases [23, 24, 25], its physiological roles in immune response and the molecular mechanisms underlying its interactions with RNA substrates remain unclear.

Here, we identified poly(A)‐specific ribonuclease (PARN) protein as a critical regulator of CSR. Genetic ablation of PARN in CSR‐competent B cells markedly impairs antibody production, especially the generation of high‐affinity isotypes. Mechanistically, PARN ensures proper post‐transcriptional processing primarily through APA to modulate the 3′ UTR length of transcripts. This function depends on its specific binding to UGUA and AA(U/A)AAA motifs, which are highly enriched in 3′ UTRs. In a mouse model of Systemic Lupus Erythematosus (SLE), PARN knockout significantly alleviates disease pathology, accompanied by reduced antibody class switching and lower serum Ig levels. Together, these findings reveal a previously unrecognized mechanism governing CSR and nominate PARN as a potential therapeutic target for mitigating pathogenic antibody responses in SLE.

## 2 Result

### 2.1 The Expression of PARN Is Increased in Systemic Lupus Erythematosus (SLE) Patients

**FIGURE 1:**
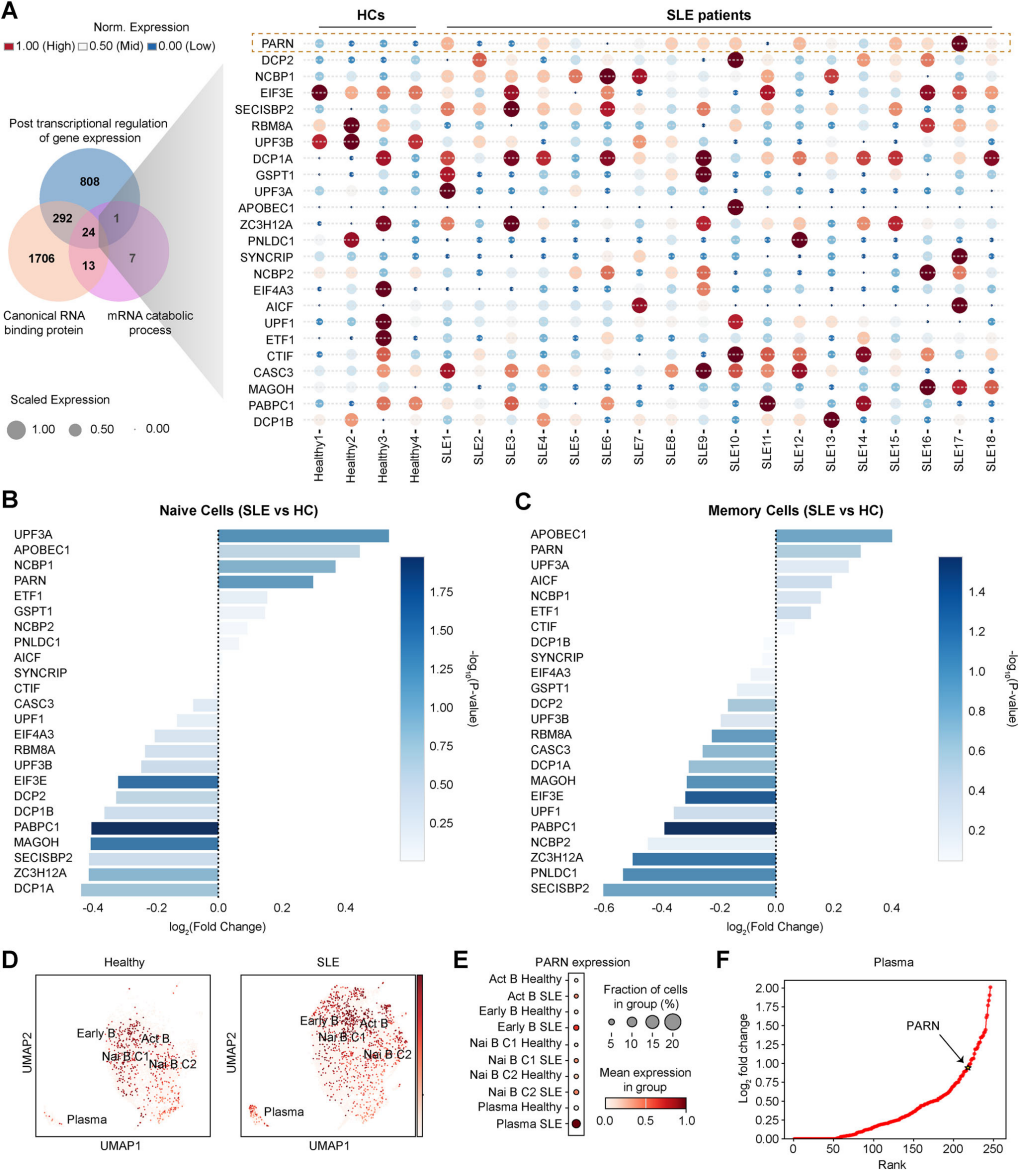
Transcriptomic quantification reveals PARN as a critical post‐transcriptional regulator potentially involved in SLE. (A) The heatmap depicts differential expression patterns of RNA‐binding protein genes in PBMCs between SLE patients (*n* = 18) and healthy controls (*n* = 4), derived from the GEO dataset (GSE122459). Dot size and color intensity within the heatmap represent normalized gene expression levels. The column of PARN expression is marked with a dashed rectangular box. (B,C) The horizontal bar chart illustrates RNA‐binding protein gene expression in naïve B cells (B) and memory B cells (C) from healthy individuals (*n* = 4) and SLE patients (*n* = 4) from the GEO dataset (GSE156751). Genes are ranked in descending order, with upregulated genes positioned toward the top and downregulated genes toward the bottom. (D,E) The expression of PARN in B cell subpopulations between healthy controls and SLE patients, displayed by a two‐dimensional UMAP plot of single cells (D), and a dot plot (E). (F) A dot plot showing the rank of RNA‐binding proteins that were upregulated in SLE patients in the RNA‐seq of plasma cells.

Systemic lupus erythematosus (SLE) is a complex autoimmune disorder characterized by multi‐organ involvement, including the skin, joints, and kidneys [26]. A growing body of evidence indicates that genetic factors result in disease susceptibility, with numerous genes implicated in the dysregulation of immune tolerance and antibody production [27]. During our previous investigation into potential regulators of SLE, we analyzed bulk RNA‐seq data (GEO: GSE122459) derived from peripheral blood mononuclear cells (PBMCs) of healthy controls (HCs) and SLE patients. By focusing specifically on a subset of genes encoding RNA‐binding proteins involved in mRNA catabolic processes and post‐transcriptional regulation of gene expression, we observed that the expression of PARN mRNA was the most pronouncedly elevated in SLE patients (Figure 1A).

Subsequently, we examined the differences in the expression of genes encoding RNA‐binding proteins between B cell subgroups (naïve B cells and memory B cells) between SLE patients and HCs by analyzing another public database of GEO: GSE156751. Significant changes were found in the expression of genes encoding RNA‐binding proteins, with *PARN*, *UPF3A*, and *APOBEC1* on the top in both naïve B cells (Figure 1B) and memory B cells (Figure 1C). Given that systemic lupus erythematosus (SLE) is a common autoimmune disease characterized by aberrant induction of type I interferons (IFNs) [28], we subsequently performed a longitudinal analysis of RNA‐binding protein expression in SLE patients over a 36‐week period during an IFN‐α kinoid (IFN‐K) clinical trial (GEO: GSE185047). A progressive decrease in the mean expression level of *PARN* mRNA was observed over time, while most genes remained unchanged or upregulated (Figure S1A).

To characterize the expression pattern of PARN in B cells at a higher resolution, we analyzed single‐cell RNA‐seq data focused on B cell subsets. Using an independent single‐cell RNA‐seq dataset (GSE135779) containing more than 200 000 PBMCs from pediatric SLE patients and matched healthy controls, B cell subsets were defined based on canonical surface markers (Figure S1B). The expression of PARN was significantly elevated in the SLE group compared with controls (Figure S1C), with more pronounced upregulation observed in plasma cell populations (Figure 1D,E; Figure S1D). In plasma cells, differential expression analysis identified over 200 RBPs that were upregulated in the SLE group; among these, PARN is highlighted as a prominent regulator of poly(A) metabolism (Figure 1F). Subsequent Gene Ontology (GO) enrichment analysis of total B cells demonstrated that pathways related to DNA repair, B cell activation, and poly(A) tail shortening were significantly enriched (Figure S1E,F), suggesting that post‐transcriptional regulation may play a critical role in modulating B cell function during SLE pathogenesis. RT‐qPCR results showed that PARN expression is significantly higher in the human peripheral blood mononuclear cell samples from SLE patients than those in healthy individuals (Figure S1G). Collectively, these findings across multiple human datasets identify PARN as a potential key regulator in the progression of SLE.

### 2.2 B Cells Require PARN for Normal Antibody Production

Since B‐cell activation is a prerequisite for effective antibody production, we examined the potential involvement of PARN in this process. First, we utilized the CH12F3 cell line, which undergoes CSR to IgA upon cytokine stimulation [29]. Three types of siRNAs were generated to knock down the expression of PARN (Figure S2A–C); PARN knockdown CH12F3 clones exhibited reduced CSR levels compared to controls (Figure S2D). Decreased concentrations of IgA were also found in the supernatant of CH12F3 cells (Figure S2E), and the post‐switched transcripts of constant α (Cα) were markedly decreased in PARN knockdown groups (Figure S2F). Of note, complementation of the FLAG‐tagged PARN expression plasmid into the *PARN* knockdown cells completely restored CSR efficiency, demonstrating that the observed defect was specifically attributable to PARN deficiency (Figure S2G–I).

**FIGURE 2:**
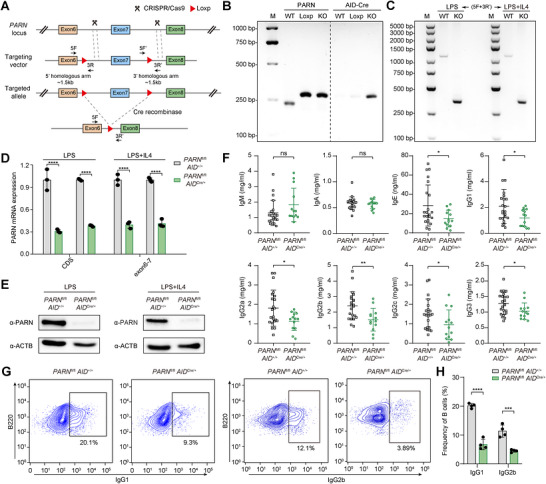
Conditional knockout of PARN in activated B cells abrogates the antibody production process. (A) Diagram of the transgenic mouse design strategy. The Cre‐LoxP system was employed to generate the PARN allele through targeted deletion of exon 7. (B) Agarose gel electrophoresis of a genotyping‐PCR to detect wild type (WT), *PARN* ^fl/fl^ *AID* ^+/+^ (LoxP) and *PARN* ^fl/fl^ *AID* ^Cre/+^ (KO) mice. (C–E) PCR analysis of genomic *PARN* (C), RT‐qPCR analysis of *PARN* transcripts (D), and immunoblotting of PARN protein (E) from *PARN* ^fl/fl^ *AID* ^+/+^ and *PARN* ^fl/fl^ *AID* ^Cre/+^ mice splenic B cells following 3 days of stimulation with LPS or LPS + IL‐4. (F) Concentrations of immunoglobulin isotypes in 7‐week‐old *PARN* ^fl/fl^ *AID* ^+/+^ and *PARN* ^fl/fl^ *AID* ^Cre/+^ mice serum. Symbols indicate biological replicates, and horizontal lines represent means. (G) Representative flow cytometry contour plots measuring CSR to IgG1 and IgG2b in splenic B cells in *PARN* ^fl/fl^ *AID* ^+/+^ and *PARN* ^fl/fl^ *AID* ^Cre/+^ mice following 3 days of stimulation with LPS or LPS + IL‐4. Numbers on plots indicate the percentage of plotted cells in the gate. (H) Proportion of *PARN* ^fl/fl^ *AID* ^+/+^ and *PARN* ^fl/fl^ *AID* ^Cre/+^ IgG1^+^ and IgG2b^+^ B cells measured by flow cytometry as shown in (G). Symbols indicate biological replicates, and bar tops represent means. ns, not significant; * *P* < 0.05; ** *P* < 0.01; *** *P* < 0.001; ns, no significance.

To further validate the impairment of CSR discovered in the PARN knockdown CH12F3 cells, the *PARN* conditional knockout (*PARN* ^fl/fl^ *AID* ^Cre/+^, cKO) mice were generated (Figure 2A,B). In this murine model, *PARN* is specifically knocked out in the antibody‐secreting cells, which highly express the activation‐induced cytidine deaminase (AID) to trigger the CSR process. In wild‐type mice, PARN was highly expressed in multiple B cell populations, particularly in mature and splenic B cells (Figure S3A,B), suggesting its important role in B cell biology. In addition, PARN was broadly expressed across immune tissues—including the spleen, thymus, and Peyer′s patches—without notable variation by sex or age (Figure S3C,D). The cKO of PARN did not affect overall body weight across age (from adulthood to ∼3 months) or sex (Figure S3E), and PARN‐deficient mice displayed normal B cell development in both the bone marrow and spleen (Figure S3F–I). Upon stimulation with immunogens like lipopolysaccharide (LPS), naïve B cells differentiate into antibody‐secreting cells. During this differentiation process in cKO mice, Cre recombinase excises the DNA segment flanked by LoxP sites within the PARN coding sequence (Figure 2C), producing an incomplete transcript which is subsequently degraded through nonsense‐mediated decay (Figure 2D,E).

Moreover, serum levels of class‐switched Ig isotypes—including IgE and several IgG subclasses—were significantly decreased in cKO mice compared with *PARN* ^fl/fl^ *AID* ^+/+^ controls (Ctrl) (Figure 2F), indicating a compromised CSR response.

### 2.3 PARN Facilitates B Cell Immune Responses Ex Vivo and In Vivo

**FIGURE 3:**
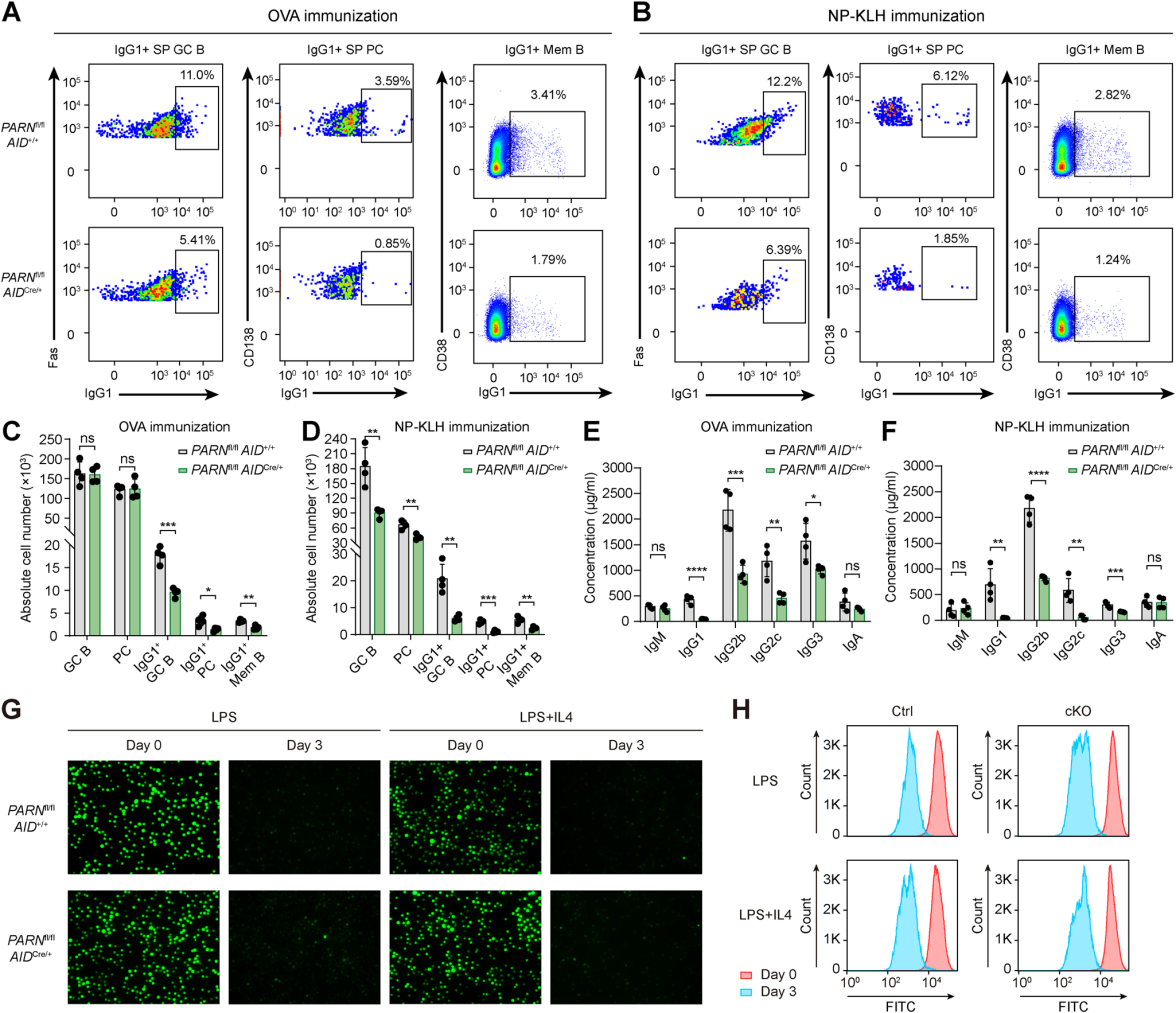
PARN is necessary for antigen‐specific B cell responses in vivo upon immunization with OVA or NP‐KLH. (A,B) Representative pseudo‐color plots from flow cytometric analysis of IgG1^+^ GC B cells (left), IgG1^+^ plasma cells (middle), and IgG1^+^ memory B cells (right) from the spleen of *PARN* ^fl/fl^ *AID* ^+/+^ (Ctrl) and *PARN* ^fl/fl^ *AID* ^Cre/+^ (cKO) mice (left margin) 2 weeks after immunization with OVA (A), and NP‐KLH (B). Numbers in outlined areas indicate the percentage of gated cells. (C,D) Bar plots showing the absolute numbers of OVA‐induced (C) and NP‐KLH‐induced (D) B cell subtypes, measured by flow cytometry as shown in (A,B). (E,F) Concentrations of immunoglobulin isotypes in OVA‐induced (E) and NP‐KLH‐induced (F) Ctrl and cKO mice serum. Symbols indicate biological replicates, and horizontal lines represent means. (G) Fluorescence displaying CFSE intensity in Ctrl and cKO splenic B cells. (H) Flow cytometric analysis for CFSE intensity shift after 3 days stimulation of LPS or LPS + IL4. * *P* < 0.05; ** *P* < 0.01; *** *P* < 0.001; **** *P* < 0.0001; ns, no significance.

*Ex vivo* stimulation of splenic B cells with LPS or LPS plus interleukin‐4 (IL‐4) demonstrated a reduced proportion of class‐switched B cell populations in the cKO group (Figure 2G,H; Figure S2J). Additionally, all post‐switched transcripts were markedly decreased in the cKO group, whereas the levels of germline transcripts (GLTs) remained unchanged (Figure S2K). To further evaluate the effect of PARN on antigen‐induced CSR in vivo, we immunized cKO and Ctrl mice with ovalbumin (OVA) or keyhole limpet hemocyanin (NP‐KLH). After two weeks immunization, in OVA‐immunized mice, the total numbers of GC B cells and plasma cells were comparable between the cKO and control groups while in NP‐KLH‐immunized mice, the cKO group showed a reduction in total GC B cell numbers and plasma cell numbers. Both cKO groups immunized with OVA and NP‐KLH exhibited significantly reduced frequencies of IgG1^+^ splenic GC B cells, IgG1^+^ plasma cells, and IgG1^+^ memory B cells (Figure 3A–D). We next quantified serum antibody composition following OVA or NP‐KLH immunization. In both immunization settings, multiple IgG subclasses were markedly decreased in the serum of cKO mice compared with their littermate controls (Figure 3E,F). To further examine whether PARN deficiency affects B cell proliferation, CFSE assays were performed. The results showed no differences in cell division following stimulation with LPS or LPS + IL‐4, indicating that PARN does not influence B cell proliferation and therefore does not indirectly contribute to increased antibody production through enhanced cellular expansion (Figure 3G,H). Taken together, we conclude that PARN is essential for CSR in B lymphocytes.

### 2.4 Regulation of the B Cell Transcriptome by PARN

To elucidate the molecular mechanisms by which PARN influences CSR in B cells, primary splenic B cells from *PARN* ^fl/fl^ *AID* ^Cre/+^ mice and their littermate controls were cultured *ex vivo*. The decrease in PARN transcript levels is shown as tracks in LPS‐stimulated splenic B cells from *PARN* ^fl/fl^ *AID* ^Cre/+^ mice compared with controls (Figure S4A). Whole‐transcriptome sequencing demonstrated high biological reproducibility between replicates within each group (Figure S4B). Differential expression analysis identified among a total of 23 066 genes, there were 752 upregulated genes and 1956 downregulated genes in the PARN knockout group (Figure S4C). Gene Ontology (GO) enrichment analysis indicated that the differentially expressed genes are primarily associated with B cell activation, Ig production, and DNA damage pathways (Figure S4D). The expression profiles of genes within the enriched pathways are illustrated by heatmaps, genomic coverage tracks, and qRT‐PCR (Figure S4E–G). These findings suggest that PARN modulates CSR in B cells through the transcriptional regulation of genes associated with Ig production.

**FIGURE 4:**
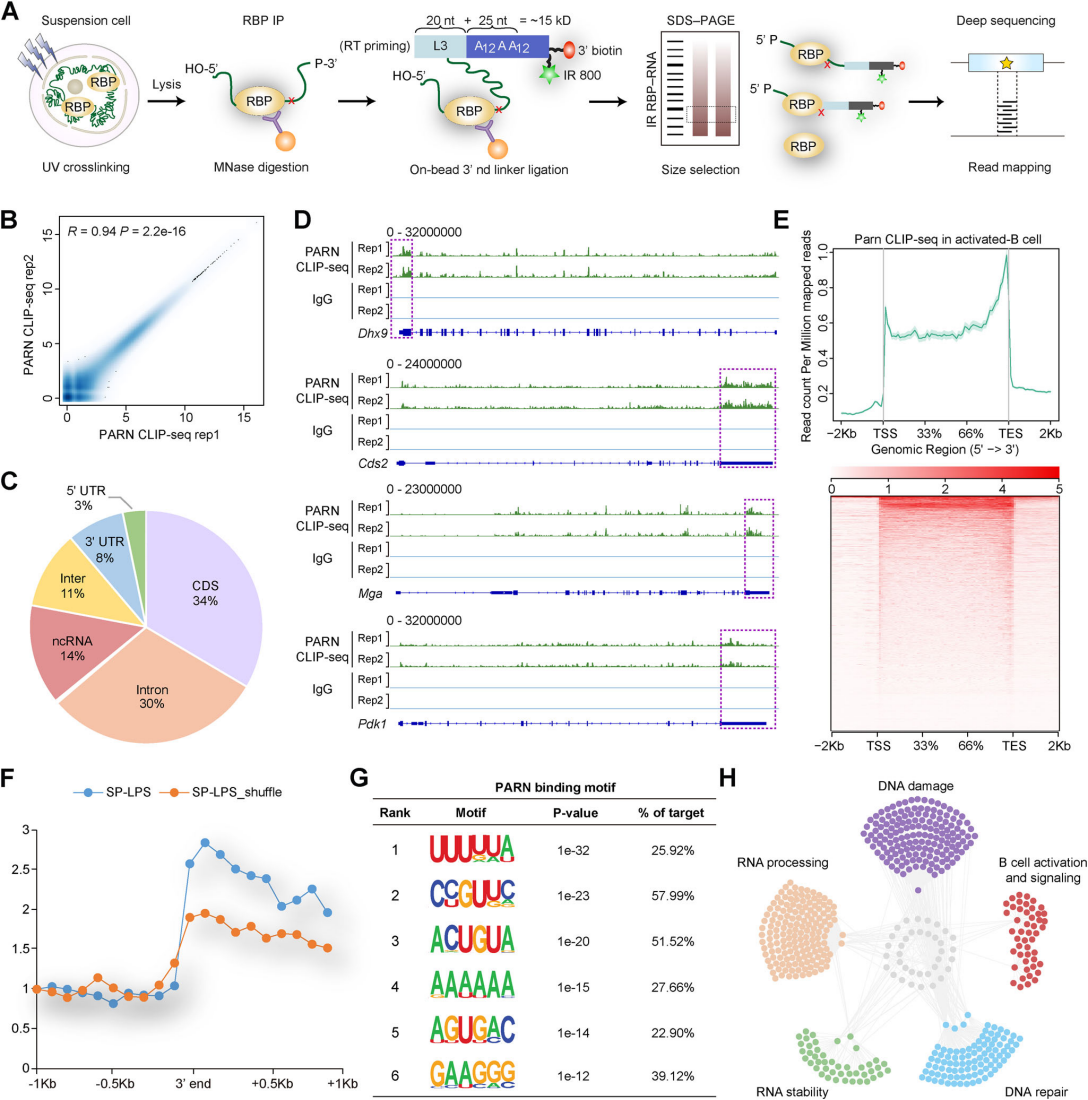
Transcriptome‐wide mapping of PARN‐binding sites reveals a preferential enrichment within 3′ untranslated regions (3′ UTRs). (A) Schematic overview of CLIP‐seq workflow. (B) Correlation between two replicates of splenic B cells following 3 days of stimulation with LPS. (C) Genomic distribution of PARN CLIP‐seq peaks. (D) The PARN‐binding peaks of transcripts. (E) Metagene analysis depicting the binding distribution of PARN across the genomic region spanning from the transcription start site (TSS) to the transcription end site (TES). (F) 3′ UTR distribution of the AAUAA motif within sequencing reads. Randomly shuffle the locations of the same size of the target motif to establish control. (G) Consensus sequence motifs in the 3′ UTR of PARN mRNA targets, as predicted by HOMER analysis. The top six enriched motifs are shown. (H) Gene ontology (GO) enrichment network showing PARN binding transcripts and their related pathways.

Although PARN is well recognized to catalyze the poly(A) tails of mRNAs, its precise binding sites and sequence‐specific binding characteristics within the transcript remain unclear. The CLIP‐seq technique allowed a detailed examination of this question (Figure 4A). Highly reproducible CLIP‐seq libraries from LPS‐stimulated splenic B cells and non‐stimulated B cells were generated (Figure 4B; Figure S5A). Analysis identified a total of 51,846 significant peaks corresponding to 7301 genes in LPS‐stimulated splenic B cells. Among these, 34% and 30% of the binding sites were located within coding sequences (CDSs) and intronic regions, respectively. The remaining PARN‐associated peaks were distributed across long non‐coding RNAs (lncRNAs, 14%), intergenic regions (11%), 3′ untranslated regions (3′ UTRs, 8%), and 5′ untranslated regions (5′ UTRs, 5%) (Figure 4C). And similar proportions are found in non‐stimulated B cells (Figure S5B). Some of the PARN‐binding tracks generated by IGV software are shown (Figure 4D; Figure S5C), and most peaks are enriched in 3′ UTRs. Meanwhile, the mapped CLIP reads to the whole genome were realigned, and the meta‐analysis revealed a strong enrichment of reads within the 3′ UTR, upstream of the transcription ending site (Figure 4E; Figure S5D).

To identify PARN‐binding preference, sequence motifs enriched in the CLIP‐seq clusters are analyzed. First, the AAUAAA motif is closely at the downstream of CDS 3′ end (Figure 4F; Figure S5E). The consensus sequence motif within 3′ UTR was UGUA and AA(U/A)AAA (Figure 4G; Figure S5F), which are known as regulatory elements in the mRNAs [30]. GO enrichment analysis further illustrates the CLIP binding genes belong to the RNA processing, RNA stability, B cell activation, and DNA repair pathways (Figure 4H; Figure S5G).

### 2.5 PARN Alters APA Events in B Cells Undergoing CSR

Alternative splicing (AS) is a well‐recognized mechanism through which RNA‐binding proteins regulate B cell activation [31, 32]. To further explore the mechanism of how PARN influences CSR, we first examined alterations in AS when PARN was knocked out. Notably, the number of AS events detected in PARN‐depleted B cell samples was relatively low, with only 132 altered AS events out of more than 30 000 total events (Figure S6A,B). In addition, the CLIP‐seq read density profiles surrounding the 5′ and 3′ splice sites were relatively flat rather than sharply peaked, and the Venn diagram revealed minimal overlap between CLIP‐seq binding genes and those exhibiting AS changes (Figure S6C,D). Combined, PARN may regulate CSR through mechanisms other than AS.

**FIGURE 5:**
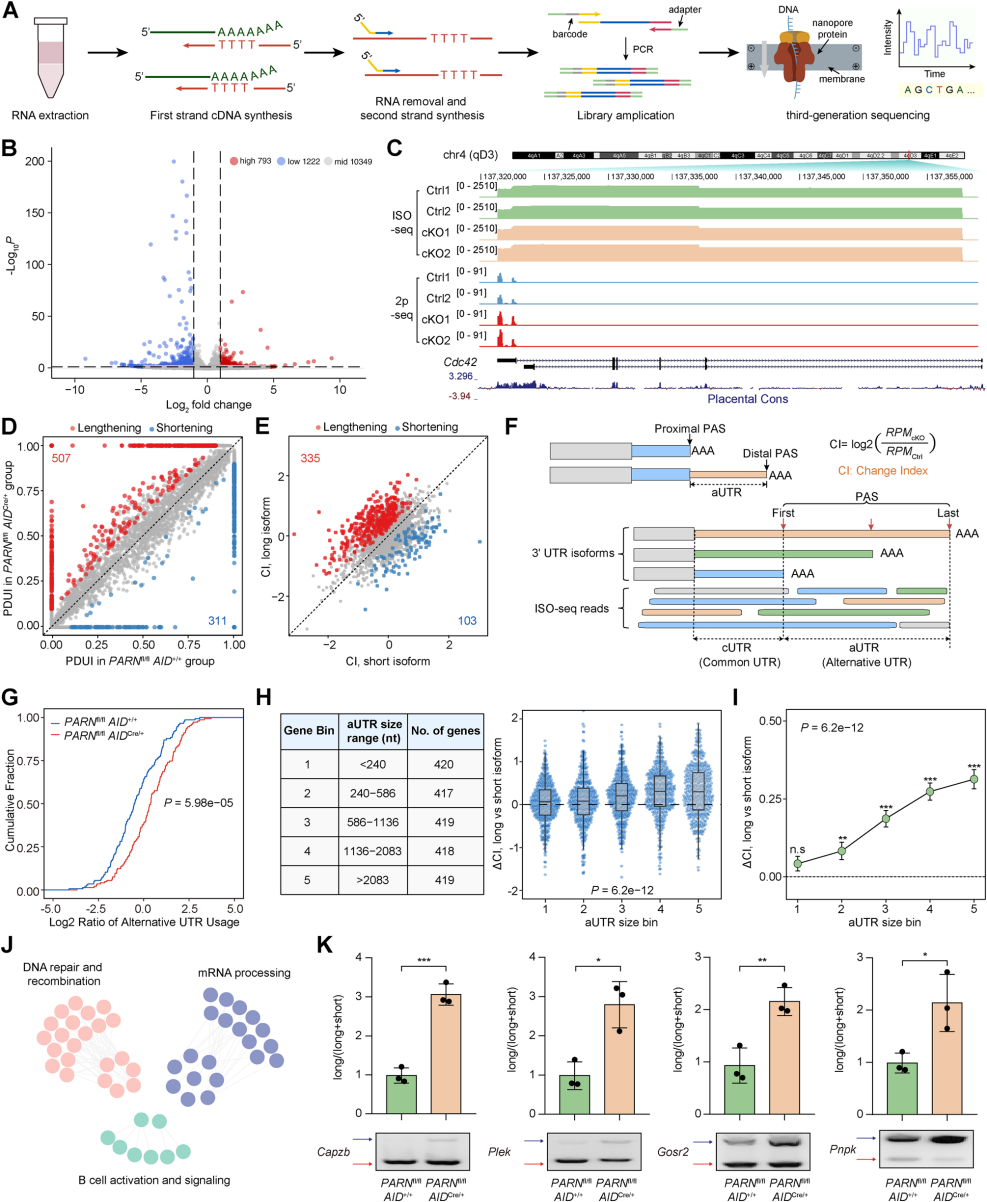
PARN regulates the 3′ UTR lengths of mRNAs through alternative polyadenylation activity. (A) Schematic workflow of ISO‐seq based on the Oxford Nanopore Technologies (ONT) platform. (B) Volcano map showing the distribution of differentially expressed transcripts from ISO‐seq data in Ctrl and cKO splenic B cells after LPS stimulation. |log2 Fold Change| ≥ 0.5. *P_adj_* ≤ 0.05. (C) Coverage tracks of Cdc42 with ISO‐seq and 2p‐seq data. Gene locus position on the top and the conservation information on the bottom. (D,E) Scatterplot comparing the percentage of distal polyA site usage index (PDUI) (D) and change index (CI) (E) of the long 3′ UTR isoform (*y*‐axis) and short 3′ UTR isoform (*x*‐axis). Genes whose isoforms have significantly different PDUI or CIs (*P* < 0.05) are highlighted in color. (F) Schematic representation of 3′ UTR size difference identification across fractions using ISO‐seq reads. The formula for the change index (CI) is shown. (G) Cumulative density curves showing the usage of alternative UTR sites in Ctrl and cKO groups. *P‐*values are based on K–S test. (H) Genes categorized into five similarly sized bins (∼420 genes) based on their alternative UTR (aUTR) size, with the size ranges provided on the left form and the difference in change index displayed on the right violin plot. (I) Relationship between aUTR size and CI difference (△*CI*) of long and short isoforms in the Ctrl and cKO groups. The *P‐*value comparing bin 1 and bin 5 genes is indicated using the Wilcoxon test. The median of each bin is plotted. Error bars indicate SEM. (J) GO enrichment analysis network showing the significantly changed genes in ISO‐seq between the Ctrl and cKO groups. (K) Alternative UTR usage of genes in Ctrl and cKO groups analyzed by PCR (*n* = 3). The ratios of transcripts with distal poly A site (blue arrow) to proximal poly A site (red arrow) are shown accordingly. *P‐*value is calculated using the Student's *t*‐test. * *P* < 0.05; ** *P* < 0.01; *** *P* < 0.001.

Considering that PARN functions as an exonuclease capable of deadenylating mRNAs at their poly(A) tails, it is plausible that PARN modulates mRNA expression by controlling the poly(A) tail length of specific transcripts. To test this hypothesis, third‐generation sequencing was performed using the Oxford Nanopore Technologies (ONT) ISO‐Seq platform (Figure 5A). Highly reproducible ISO‐seq libraries from LPS‐stimulated splenic B cells in cKO and Ctrl mice were generated (Figure S7A,C). More than 10 000 transcripts with different poly(A) sites were identified together with their expression levels (Figure 5B; Figure S7B). Among these, 507 genes exhibited increased usage of distal poly(A) sites—reflected by a higher percentage of distal poly(A) site usage index (PDUI) values—in cKO mice compared with the Ctrl mice. Cdc42, for example, is a targeted gene which has an overall elevated expression in the cKO group, and the abundance of the long transcript is upregulated as well (Figure 5C). Notably, this number exceeded the set of genes preferentially expressing longer 3′ UTR isoforms in the Ctrl group (Figure 5D). The change index (CI), which quantifies isoform abundance on a log scale, revealed that cKO B cells preferentially retained long 3′ UTR isoforms over short ones (Figure 5E,F). This global shift toward distal poly(A) site usage was further supported by cumulative distribution analysis, in which the curve for the cKO group was shifted to the right of that of the Ctrl group, indicating a higher mean alternative UTR (aUTR) usage in cKO cells (Figure 5G).

To investigate how 3′ UTR length influences expression changes, genes with APA were grouped into five bins of comparable size (∼420 genes each) based on their aUTR length (Figure 5H). We observed a positive correlation between aUTR length and the magnitude of CI change (ΔCI), such that genes with longer 3′ UTRs exhibited greater increases in long isoform expression in the cKO group (Figure 5I). We also performed 2p‐seq, which uses oligo‐dT and T20VN primers to capture and amplify transcripts with diverse poly(A) tail lengths [33], to characterize APA events (Figure S8A–D). This approach enables to display the peaks of read distributions spanning from proximal to distal poly(A) sites, thereby visualizing the relative abundance of alternative 3′ UTR isoforms for each gene (Figure S8E). GO enrichment analysis demonstrates that genes with lengthening 3′ UTR are enriched in the RNA processing, B cell activation, and DNA recombination pathways (Figure 5J). APA events of these genes are validated by RT‐PCR (Figure 5K). In the gene set enrichment analysis (GSEA), the mRNA catabolic process pathway was downregulated when transcripts with 3′ UTR alterations were analyzed (Figure S7D). Furthermore, analysis of poly(A) tail length revealed a global increase in poly(A) length in the cKO group (Figure S7E). Among transcripts with lengthened 3′ UTRs, a clear shift was observed toward longer poly(A) tails, exhibiting approximately a onefold increase relative to the Ctrl group (Figure S7F,G). Collectively, PARN transcriptome‐wide shapes 3′ UTR architecture, predominantly eliciting the selection of shorter 3′ UTR isoforms by hydrolyzing poly(A) tails, through which process it reconfigures the expression of CSR‐associated genes.

### 2.6 PARN Enables the Intramolecular Connections Within Transcripts in UGUA and Poly(A) Motifs

**FIGURE 6:**
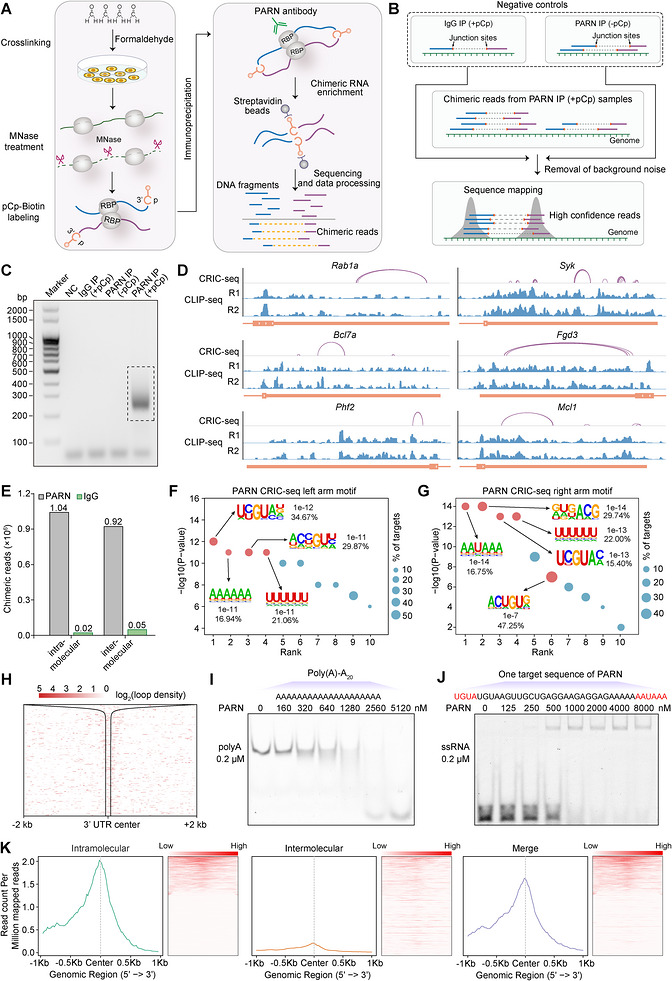
PARN binds simultaneously to both the UGUA enhancer and the AAUAAA polyadenylation signal within the 3′ UTR. (A) Schematic workflow of CRIC‐seq. (B) CRIC‐seq data analysis pipelines. (C) Size distribution of CRIC‐seq libraries. (D) CRIC‐seq tracks represent PARN‐mediated interaction within the same transcript (purple loops indicate the interactions). (E) Bar plot showing the millions of high‐confidence intra‐ and inter‐molecular chimeric reads detected by PARN CRIC‐seq. (F,G) Consensus PARN‐binding motifs around the 3′ UTR PARN CRIC‐seq loops including left arm motifs (F) and right arm motifs (G) using HOMER analysis. The top six enriched motifs are shown. (H) The aggregation plot showing PARN‐associated intramolecular loops confined within the 3′ UTR center, ranked based on their length. The black curve indicates the boundary of 3′ UTR. Color intensity indicates CRIC‐seq read density. (I) The electrophoretic mobility shift assay (EMSA) of the PARN‐catalyzed A_20_ shortening process. A_20_ (0.2 µm) was incubated with different concentrations of PARN protein (0, 160, 320, 640, 1280, 2560, and 5120 nm) for 10 min at 37°C. (J) EMSA of PARN‐triggered UGUA and AAUAAA motif binding process. Single‐stranded RNA (ssRNA) with PARN‐binding motifs (0.2 µm) was incubated with different concentrations of PARN protein (0, 125, 250, 500, 1000, 2000, 4000, and 8000 nm) for 15 min at 30°C. (K) Metagene profile illustrating the overlapping area between PARN CLIP‐seq and CRIC‐seq data, as shown in intramolecular (left), intermolecular (middle), and merged (right) fragments with 3′ UTR, ordered in descending 3′ UTR length. Color intensity indicates the overlapping density.

There are many regulatory elements located in the 3′ UTR, and our data represent that UGUA ranks the top consensus motif in our CLIP‐seq data. Although it is known to function as an upstream element for APA [34], the detailed mechanism has not been fully clarified. We therefore sought to determine how the concurrent binding of PARN to UGUA and other *cis*‐regulatory sequences elicits APA. To this end, we implemented CRIC‐seq to characterize the proximal RNA interactome associated with PARN and reveal the spatial contacts guiding its function (Figure 6A). Using lysates from LPS‐stimulated B cells, two biological replicates of CRIC‐seq libraries were generated for the PARN IP (+pCp) samples, along with negative controls (IgG IP (+pCp) and PARN IP (‐pCp)) (Figure 6B; Figure S9A). A distinct smeared band ranging from 200 to 500 bp was detected in the PARN IP (+pCp) samples, indicative of successful library construction (Figure 6C). After purification of the target bands for deep sequencing, approximately 1.04 million chimeric reads corresponding to PARN IP (+pCp) intramolecular RNA–RNA contacts were obtained, representing a slightly higher proportion than that of intermolecular contacts (Figure 6E).

Furthermore, the composition of intramolecular interactions was analyzed and found to be enriched between introns and 3′ UTRs (Figure S9B). Given the relatively small proportion of 3′ UTRs in the genome length‐wise, this enrichment suggests that PARN preferentially promotes RNA–RNA interactions within 3′ UTRs. CRIC‐seq tracks uncover numerous looping structures corresponding to 3′ UTR interactions (Figure 6D). The overall loop length is mainly below 1Kb (Figure S9C). Sequence motif analysis of RNA loops identified significant enrichment of UGUA and AA(U/A)AAA motifs (over 34% and 16% of all targets, respectively) in both the left and right arms (Figure 6F,G), and most loops are contained within the 3′ UTRs (Figure 6H).

To investigate the biochemical activity of PARN, catalytic and binding assays were performed. The results demonstrated that poly(A) single‐stranded RNA was progressively shortened with increasing concentrations of PARN, consistent with previously findings [35] (Figure 6I). PARN was confirmed to specifically bind single‐stranded RNAs containing UGUA and AA(U/A)AAA motifs (Figure 6J). In addition, a high degree of overlap was observed between CLIP‐seq and CRIC‐seq binding sites across the genome, particularly within the 3′ UTRs (Figure 6K; Figure S9D). This result provides reciprocal validation between the CLIP‐seq and CRIC‐seq datasets, reinforcing the reliability of the identified RNA–protein and RNA–RNA interaction sites. In summary, our data indicate that PARN promotes the spatially coordinated binding of UGUA and AAUAAA motifs. Since poly(A) sites are positioned adjacent to the poly(A) tail, this configuration is also instrumental in enabling PARN‐mediated poly(A) tail hydrolysis.

### 2.7 PARN Regulates *Foxp1* Isoform Balance Through Alternative Polyadenylation

**FIGURE 7:**
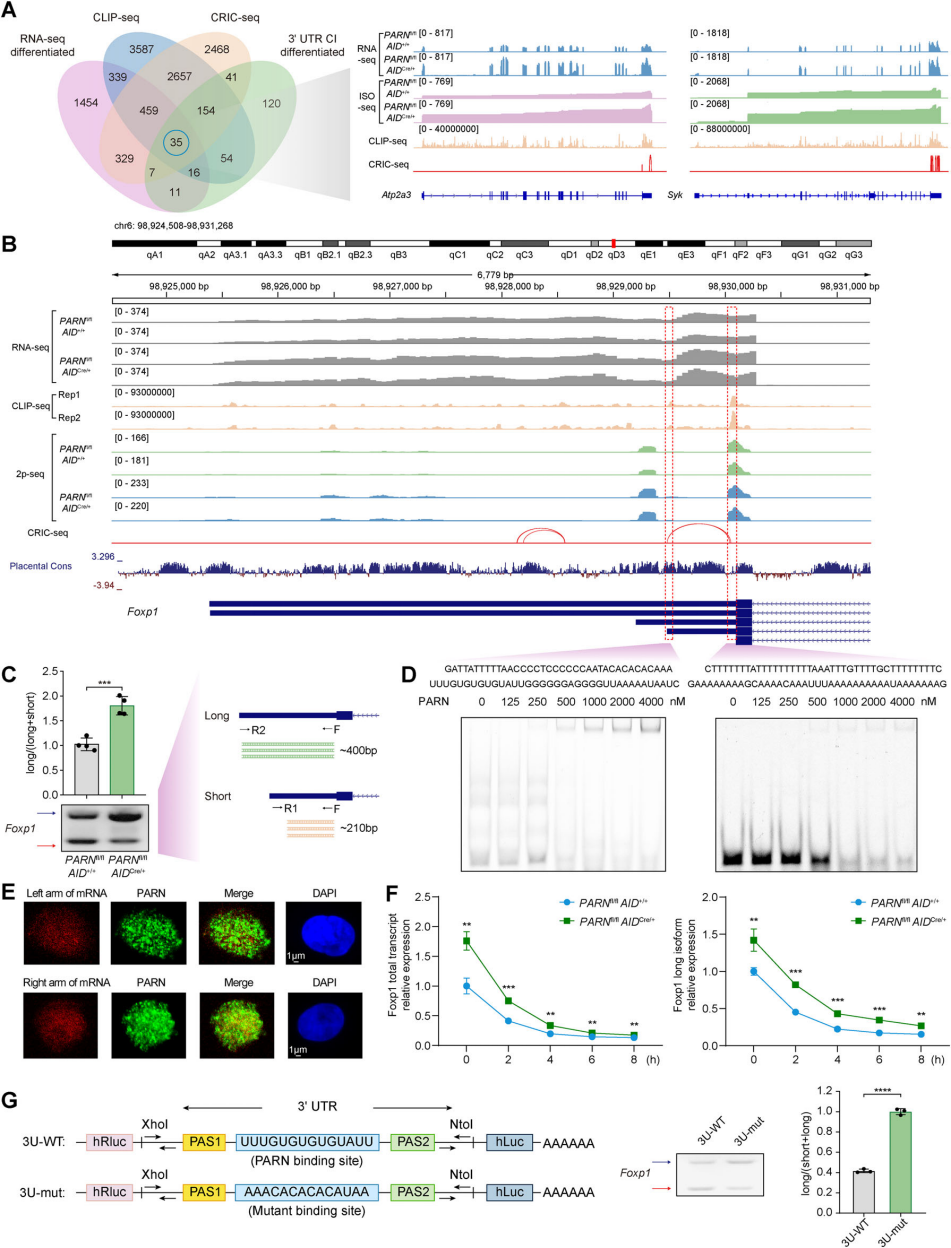
FOXP1 serves as the downregulation factor of PARN in the process of CSR. (A) Venn diagram revealing 36 transcripts with CLIP‐seq and CRIC‐seq binding sites, downregulated at the mRNA level, and differentiated 3′ UTR isoforms (left). IGV tracks of two representative genes from the overlapping set are shown (right). (B) Coverage tracks of the *Foxp1* transcript, including RNA‐seq, CLIP‐seq, 2p‐seq, CRIC‐seq tracks. (C) RT‐PCR showing the lengthened isoforms of *Foxp1* in the *PARN* ^fl/fl^ *AID* ^Cre/+^ (cKO) splenic B cells compared to those of *PARN* ^fl/fl^ *AID* ^+/+^ (Ctrl). Bar plot displaying the intensity of two *Foxp1* isoforms (left) and IGV track illustrating the isoform location, respectively (right). (student *t*‐test, *** *P* < 0.001, *n* = 4) (D) Electrophoretic mobility shift assay showing single‐stranded RNA (ssRNA) binding activities of PARN across a concentration gradient of PARN with 0.2 µm ssRNA. (E) Fluorescence in situ hybridization of PARN and RNA sequence from *Foxp1* transcript, and the nucleus stained with DAPI. Scale bar, 1 µm. (F) mRNA decay curves depicting transcript stability in LPS‐stimulated B cells from Ctrl and cKO groups after actinomycin D treatment (measured at 2‐hour intervals). *P*‐value is adjusted by multiple *t*‐test using false discovery rate (FDR). (G) RT‐PCR showing the lengthened isoforms of *Foxp1* in 293T cells transfected by PARN plasmid and *Foxp1* 3′ UTR (3U‐WT) or 3′ UTR mutant (3U‐mut) plasmid. (student *t*‐test, **** *P* < 0.0001, *n* = 3).

Next, we delve into the molecular mechanisms through which PARN influences CSR regulation. By integrating multiple sequencing datasets, we constructed a Venn diagram illustrating the overlap among differentially expressed genes between cKO and Ctrl groups, PARN‐bound targets identified by CLIP‐seq and CRIC‐seq, and transcripts exhibiting APA alterations (Figure 7A). From this intersection, a group of genes, such as *Foxp1*, *Syk*, and *Atp2a3*, have the following features: the transcripts are upregulated in cKO cells; possess differentiated APA events in 3′ UTR isoforms; are directly bound by PARN; have spatially interactions within 3′ UTR (Figure 7B). Among these genes, the post‐switched antibody transcripts (γ1, γ2b, γ3, ε) neither exhibited detectable APA alterations nor showed evidence of direct binding by PARN. This observation prompted us to search for additional PARN‐targeted genes that might influence CSR.

Within the aforementioned intersected gene set, *Foxp1* emerged as a compelling candidate, as it has been previously reported to function as a negative regulator of CSR [36]. Given its known role and its top‐ranked position in highly expressed transcripts (Figure S10A), we first knocked down of *Parn* or *Foxp1* or both genes in CH12F3 cells, and the CSR process was rescued when *Foxp1* was silencing (Figure S10B,C), proving that *Foxp1* is one of PARN′s downstream regulators. Next, we focused on elucidating how PARN regulates *Foxp1* expression through APA modulation. RT‐PCR confirmed a preferential shift toward the long 3′ UTR isoform of *Foxp1* in the cKO group (Figure 7C), consistent with a higher abundance of longer poly(A) length calculated by read density (Figure S10D). Moreover, PARN binds both the left and right arms of the *Foxp1* 3′ UTR—regions enriched for UGUA and AAUAAA motifs (Figure 7D,E). Functional assays further demonstrated that PARN can hydrolyze transcripts at both arms, whereas transcripts capped with a 3′ phosphate remain bound by PARN but are resistant to hydrolyzation (Figure S10E,F). Considering that, in physiological conditions, UGUA and AAUAAA motifs are followed by additional downstream sequences, including the poly(A) tail, these findings suggest that PARN recognizes these motifs to localize to the terminal region of the transcript, where it subsequently exerts its catalytic activity to degrade the poly(A) tails.

The decay rate of *Foxp1* transcript was examined by treating splenic B cells with actinomycin D (Act D), a fast‐acting DNA intercalator that inhibits RNA polymerase II (RNAPII) [37], after 3 days of LPS stimulation. For the total 3′ UTR transcripts, the initial expression level of *Foxp1* in the cKO group was approximately twice that in the Ctrl group. In particular, after 8 h of Act D treatment, both the total *Foxp1* transcripts and the long isoforms exhibited significantly faster mRNA decay rates in cKO cells compared to Ctrl cells (Figure 7F). To further validate that PARN directly regulates APA of *Foxp1*, we mutated the UGUA motif within its 3′ UTR. Compared with the wild‐type construct (3U‐WT), the mutant (3U‐mut) exhibited a significantly increased proportion of the long isoform (Figure 7G). In addition, we generated a plasmid encoding the catalytically inactive PARN mutant (D28A). Expression of this mutant led to lengthening of Foxp1 transcripts (Figure S10G). Consistent with previous reports [38], this mutation impaired both the RNA‐binding activity and deadenylase function of PARN (Figure S10H). CRIC‐seq data suggested that PARN interacts with an intramolecular RNA loop formed within the *Foxp1* 3′ UTR. Disruption of this loop by mutating each arm resulted in a significant shift of *Foxp1* APA toward the long isoform. Importantly, restoring base‐pairing interactions through compensatory mutations rescued normal APA patterns (Figure S10I).

Together, these results indicate that PARN preferentially recognizes transcripts harboring UGUA and AAUAAA motifs. *Foxp1*, which contains alternative poly(A) sites and PARN‐targeted sequence elements, is specifically regulated by PARN. Through its deadenylase activity, PARN modulates *Foxp1* expression by controlling the selection and stability of its 3′ UTR isoforms.

### 2.8 PARN Knockout in Antibody‐Secreting Cells Alleviates the Phenotype of SLE

**FIGURE 8:**
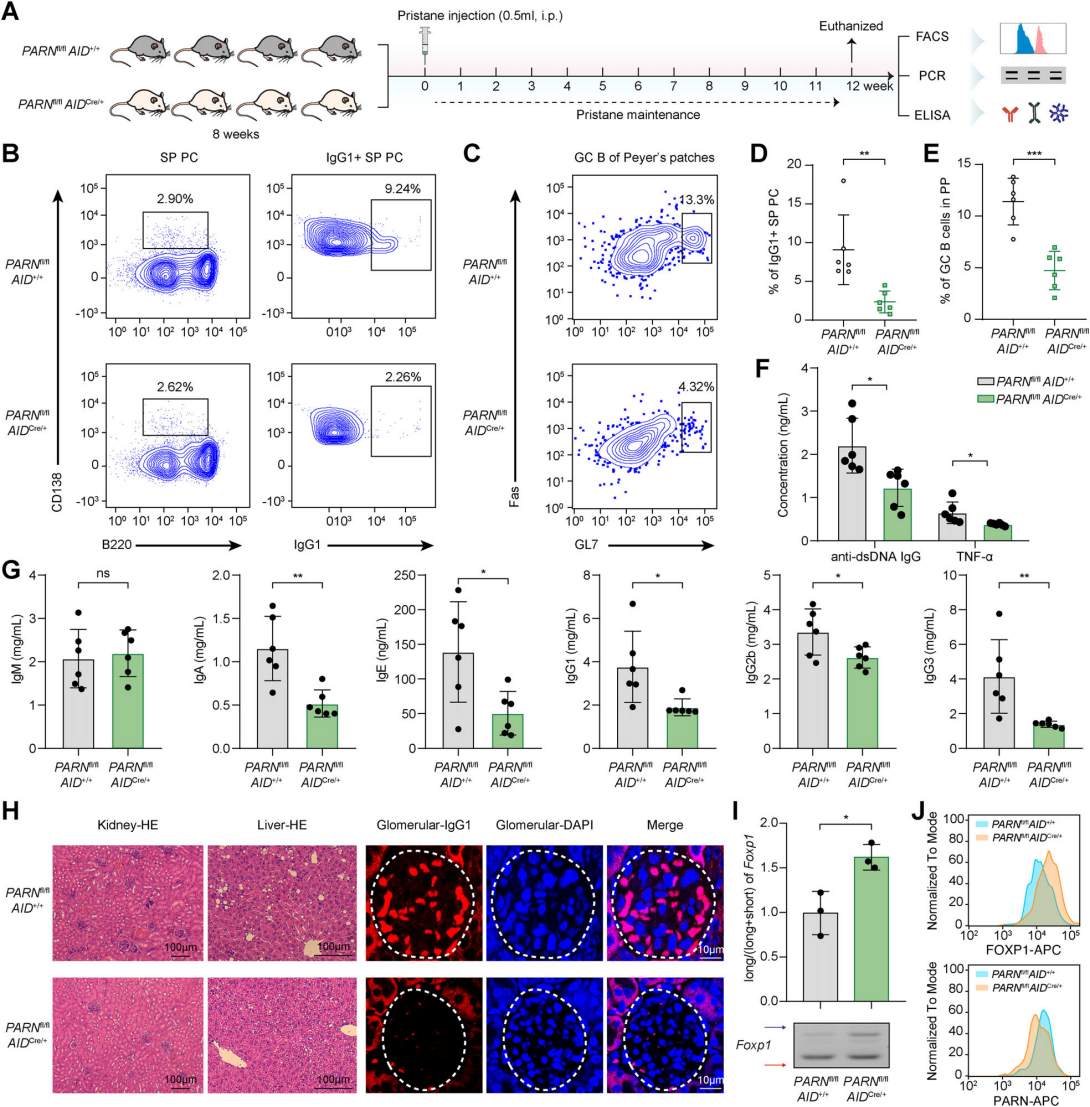
Targeted knockout of *Parn* in antibody‐secreting cells alleviates lupus‐like phenotypes in the SLE mouse model. (A) Overview of the pristane‐induced SLE mouse model strategy. (B,C) Representative contour plots from flow cytometric analysis of splenic IgG1^+^ plasma cells (B), and germinal center (GC) B cells (C) of *PARN* ^fl/fl^ *AID* ^+/+^ (Ctrl) and *PARN* ^fl/fl^ *AID* ^Cre/+^ (cKO) mice 8 weeks after intraperitoneal injection with pristane. (D,E) Bar plots showing the proportions of splenic IgG1^+^ plasma cells (D) and GC B cells (E), measured by flow cytometry as shown in (B,C). (F,G) Concentrations of anti‐double‐stranded DNA IgG and tumor necrosis factor‐alpha (TNF‐α) (F) and different immunoglobulin isotypes (G) in pristane‐induced *PARN* ^fl/fl^ *AID* ^+/+^ and *PARN* ^fl/fl^ *AID* ^Cre/+^ mice serum. Symbols indicate biological replicates, and horizontal lines represent means. (student *t*‐test, * *P* < 0.05, ** *P* < 0.01, ns, no significance, *n* = 6) (H) Histopathological analysis of kidney and liver tissues by H&E staining and glomerular IgG deposition analysis by immunofluorescence staining. (I) PCR analysis of different usage of *Foxp1* isoforms in cKO and Ctrl groups. (J) Flow cytometric analysis of PARN and FOXP1 protein expression in the cKO and Ctrl groups.

Building on the aforementioned findings that establish PARN′s correlation with SLE and its role in antibody production, we next sought to test its direct pathological role in SLE pathogenesis by generating a pristane‐induced lupus mouse model (Figure 8A). Twelve weeks after intraperitoneal pristane injection, both splenic IgG1^+^ plasma cells and GC B cells in PP were significantly reduced in the cKO group compared with the Ctrl group (Figure 8B–E). Serum levels of double‐stranded DNA and TNF‐α were also decreased in cKO mice (Figure 8F). Analysis of serum Ig further revealed that multiple antibody isotypes—including IgG subclasses, IgA, and IgE—were significantly lower in cKO mice than in their Ctrl counterparts (Figure 8G), suggesting that PARN deficiency mitigates key immunopathological features associated with SLE. Histopathological analysis showed that in cKO mice, the inflammatory infiltration was lessened in both kidney and liver tissues; the IgG1^+^ deposition in the glomerular region was also decreased in cKO mice (Figure 8H). In accordance with the aforementioned molecular mechanism, cKO GC B cells demonstrated preferential utilization of the long *Foxp1* isoform (Figure 8I), accompanied by an increased level of FOXP1 protein (Figure 8J).

Human samples provide more evidence that PARN is relevant to disorders with malfunction of antibody production. Transcriptomic analyses of peripheral blood samples from individuals with rheumatoid arthritis (GSE93272) or SLE (GSE61635) showed significantly elevated PARN expression in both autoimmune conditions (Figure S11A,B). These findings collectively implicate PARN dysfunction—either through loss‐of‐function mutations or pathological overexpression—in human diseases involving dysregulated antibody production.

### 2.9 PTBP1 Cooperates With PARN in the Process of CSR

**FIGURE 9:**
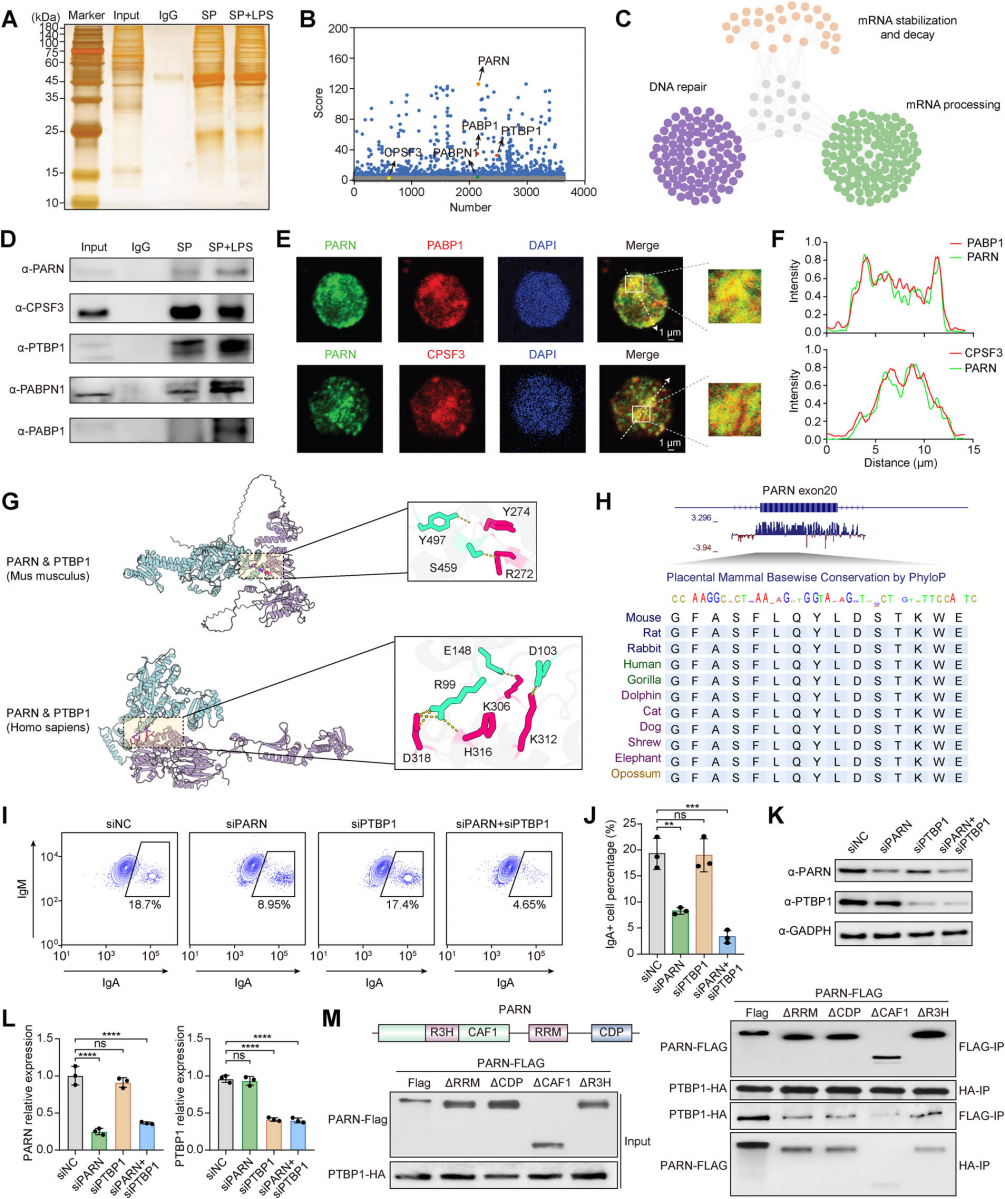
PARN recruits co‐working proteins in the process of CSR. (A) Silver‐staining of PARN immuno‐precipitates from LPS‐stimulated splenic B cells. (B) Scatter plot of protein scores demonstrating MS‐detected proteins between two groups of parallel mixed samples. (C) Network showing GO enrichment analyses of PARN‐binding proteins. (D) An immunoprecipitation (IP) experiment was performed in spleen B cells extracts after LPS stimulation. (E,F) Co‐immunostaining (E) and intensity scan map (F) of the CH12F3 cell was performed using PARN and PARN‐binding proteins (PABP1 and CPSF3) antibodies. DNA was stained with DAPI. Scale bar, 1 µm. (G) Structural prediction of the interaction site of PARN and PTBP1 proteins in the species of *Mus musculus* and *Homo sapiens* using AlphaFold 3. (H) Conservation analysis of amino acid sequences in PARN across different species. (I) Representative contour plots from flow cytometric analysis of IgA^+^ cells in CH12F3 cell lines following transfection with NC, siPARN, siPTBP1, and siPARN+siPTBP1. (J) Bar plots showing the proportions of IgA^+^ cells, measured by flow cytometry as shown in (I). (K,L) Immunoblotting (K) and RT‐qPCR (L) analysis of PARN, PTBP1 expression levels in CH12F3 cell lines following transfection with NC, siPARN, siPTBP1, and siPARN+siPTBP1. The expression levels were normalized to *Gapdh* transcripts and the NC group in quantitative PCR analysis (*n* = 3, mean ± SD). (M) Immunoblot analysis of FLAG‐tagged PARN and HA‐tagged PTBP1 following co‐immunoprecipitation, including assays using various truncation mutants of PARN. A schematic of several PARN domains is shown.

In many biological processes, proteins function cooperatively within multiprotein complexes to execute sophisticated regulatory tasks. Based on this principle, we sought to identify cofactors that interact with PARN during CSR regulation. We performed immunoprecipitation (IP) of PARN from splenic B cells with LPS stimulation (Figure 9A). Mass spectrometric (MS) analysis discovered a set of PARN‐interacting proteins that were enriched in DNA repair, mRNA‐processing, and mRNA stabilization pathways, including PTBP1 and PABP (Figure 9B,C). It is also observed that the abundance of PTBP1 and PABP increased following LPS stimulation (Figure 9D). Using structural prediction and immuno‐colocalization approaches, we visualized the PARN interactions with PABP, CPSF3, and PTBP1 (Figure 9E,F). The predicted PARN–PTBP1 binding interface displayed a high degree of sequence conservation across species (Figure 9G,H; Figure S12E).

We further examined the impact of PARN with PTBP1 or PABP on CSR efficiency in the CH12F3 cell line following knockdown of PARN with PTBP1 or PABP. Loss of PARN and PTBP1 significantly reduced the proportion of IgA^+^ cells, and co‐knockdown of PARN and PABP showed a similar impairment (Figure 9I–L; Figure S12A–D). To delineate the PARN domains required for cofactor binding, we generated a panel of PARN deletion mutants (ΔCAF1, ΔR3H, ΔRRM, and ΔCDP) in the pcDNA3.0 vector (Figure 9M). IP assays demonstrated that PTBP1‐HA interacted strongly with full‐length of PARN, but its binding to ΔCAF1 and ΔCDP was markedly weakened, indicating that these domains are essential for PARN‐cofactor interactions.

## 3 Discussion

**FIGURE 10:**
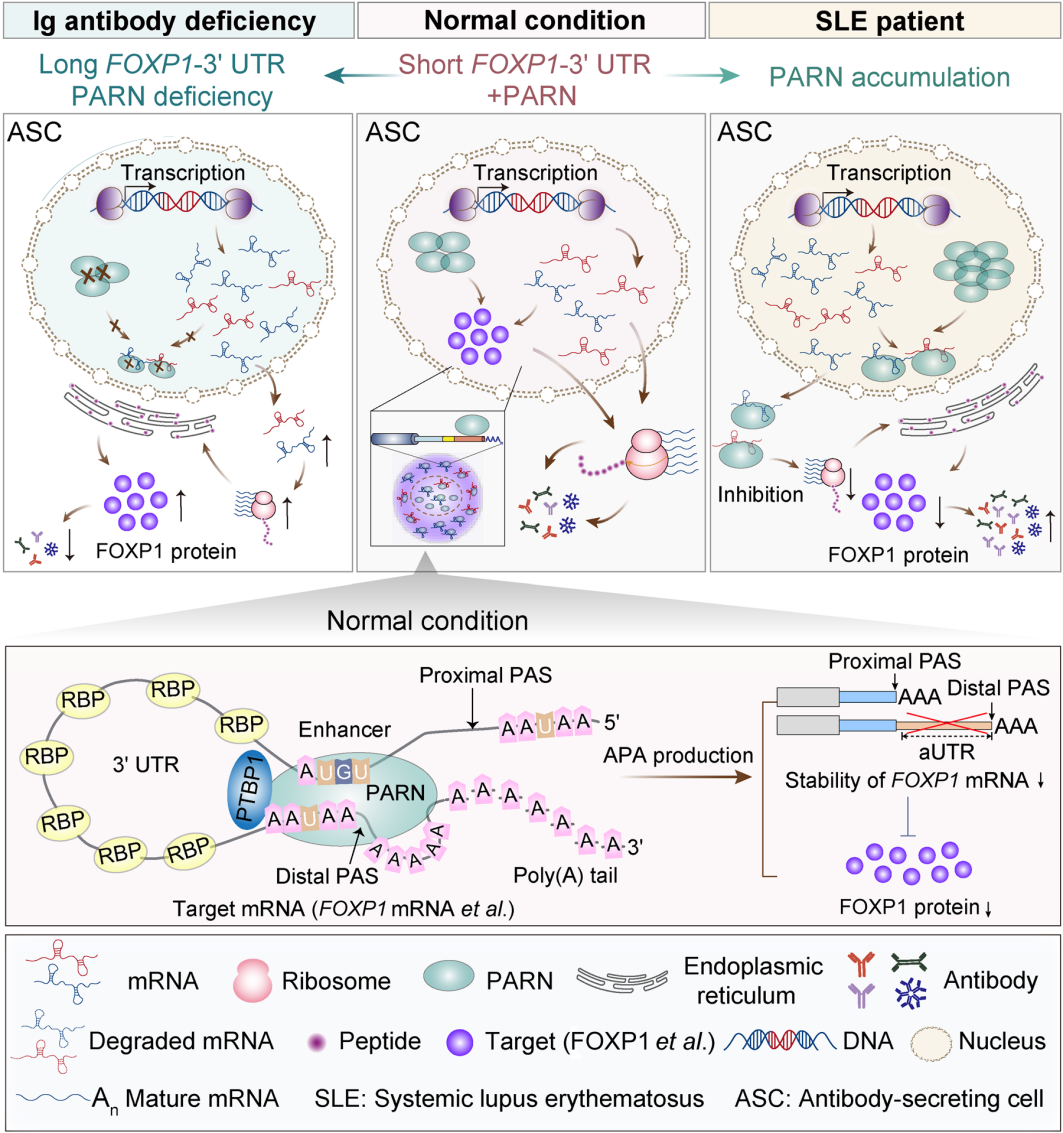
Proposed model illustrating how PARN regulates CSR in antibody‐secreting cells (ASCs). In ASCs, PARN recruits PTBP1 to form a protein complex that binds to UGUA and AAUAAA motifs on target RNAs. This sequence‐specific binding enables PARN to globally modulate 3′ UTR length through alternative polyadenylation (APA). *Foxp1* is one such PARN‐regulated transcript: in ASCs, the abundance of its long 3′ UTR isoform is reduced, whereas shorter 3′ UTR isoforms with less stability are preferentially produced. This shift toward less stable isoforms lowers overall *Foxp1* expression, thereby facilitating class switch recombination (CSR). In PARN‐deficiency mice, reduced PARN activity permits an increased usage of the long 3′ UTR isoform of *Foxp1*, leading to elevated protein expression and consequent suppression of CSR. Conversely, PARN is highly expressed in SLE patients, which results in diminished *Foxp1* levels and drives ASCs toward excessive antibody production.

PARN is a deadenylase that hydrolyzes adenosine monophosphate at the RNA 3′ end, thereby shortening the poly(A) tail [39]. It has been implicated in diverse biological processes, including cell self‐renewal [22], stabilizing microRNAs [40], and telomere maturation [25]; however, the elaboration on the molecular mechanism is somewhat insufficient. Here, we demonstrate for the first time that PARN acts as an RNA‐binding protein and a pivotal global regulator of CSR through APA. To study its function specifically in activated B cells, we employed an AID‐Cre transgenic mouse model [41] and generated *PARN* ^fl/fl^ *AID* ^Cre/+^ mice. Results show that elevated PARN expression promotes CSR, whereas PARN deficient shifts 3′ UTR usage toward longer isoforms. Transcriptome‐wide analyses reveal that PARN preferentially recognizes UGUA and AAUAAA motifs‐regulatory elements enriched in 3′ UTRs‐and spatially coordinates their interactions. Among its targets, *Foxp1* is directly regulated by PARN: loss of PARN increases the abundance of the long isoform of *Foxp1*, upregulates protein Foxp1 expression, and ultimately suppresses CSR. Furthermore, PARN interacts via its catalytically active CAF1 domain with cofactors such as PTBP1, forming a protein complex that contributes to CSR modulation. In summary, our study proposes a mechanistic model in which PARN orchestrates APA‐dependent transcriptome remodeling to control the production of high‐affinity antibodies (Figure 10).

Studies have established that RBPs regulate CSR through multiple mechanisms, including guiding AID localization to Ig loci [15], modulating the 3′‐end processing of GLTs [42], or mediating transcriptional pausing [43]. However, whether APA contributes to the efficiency or specificity of CSR remains largely unexplored and represents a critical unanswered question in B‐cell biology. For the first time, we demonstrated that PARN globally binds 3′ UTRs within mRNAs to regulate APA instead of just shortening the poly(A) tails. By integrating CLIP‐seq with CRIC‐seq in activated B cells, we defined both the sequence and spatial binding landscape of PARN, revealing its widespread 3′ UTR interactions. We show that PARN binds UGUA and AA(U/A)AAA motifs to architect RNA spatial structures that facilitate APA. Although the UGUA motif is known to recruit CFIm to enhance mRNA processing [44], and motif spacing has been linked to poly(A) site (PAS) usage [45], the detailed mechanism of how these motifs influence gene expression is rarely clarified. Our ISO‐seq data demonstrate that APA orchestrated by PARN serves as a direct mechanism of gene regulation, moving beyond the conventional view of 3′ UTR length preference to include expression‐level control. In detail, loss of PARN results in widespread 3′ UTR lengthening, reflecting a shift toward preferential usage of distal PASs. Notably, the degree of 3′ UTR extension correlates positively with the magnitude of transcript upregulation in PARN‐deficient B cells.

Our work demonstrated that PARN normally recognizes *cis*‐elements for targeting and shortens poly(A) tails through its nuclease activity. Also, PARN can hydrolyze the fragments of RNAs when the last nucleotide is not adenosine. For instance, *Foxp1*, the gene with the top‐ranked number of transcripts, is highly expressed in B cell development while downregulated in germinal center B cells for CSR; when maintaining at high expression levels, it suppresses γ1 germline transcription and impedes switching to the IgG1 isotype [36]. We found that PARN functions as an upstream regulator that represses *Foxp1* expression during CSR. PARN specifically binds UGUA and AAUAAA motifs within the 3′ UTR of *Foxp1* and hydrolyzes its poly(A) tail. Upon PARN knockout, the capacity of B cells to produce high‐affinity antibodies is diminished. Although APA has emerged as a widespread phenomenon modulating gene expression, previous studies have primarily focused on the molecular machinery governing cleavage and polyadenylation rather than deadenylation [46]. Our study addresses this gap by elucidating regulatory processes acting downstream of poly(A) tail addition.

In the pathogenesis of SLE, the majority of cases exhibit a polygenic inheritance pattern [47], and due to its pronounced heterogeneity, diagnosis remains predominantly clinical and continues to pose considerable challenges. RBPs have been proven to be biomarkers for many autoimmune diseases. For instance, single‐nucleotide polymorphisms (SNPs) in TTP, an RBP with a CCCH zinc finger‐containing domain, have been linked to the duration and risk of rheumatoid arthritis in diverse populations [48, 49]. Similarly, the adenosine‐to‐inosine RNA editor ADAR1 is implicated in rheumatoid arthritis [50], and its mutations cause Aicardi–Goutières syndrome (AGS) [51]. Besides, mutations in 3′ repair exonuclease 1 (TREX1) are also associated with AGS and SLE [52], highlighting a wide range of post‐transcriptional control of RBPs in immune homeostasis. Our study identified a broad upregulation of PARN in SLE patients relative to healthy controls, especially in plasma cells. This phenomenon is validated by the following SLE mouse model. This finding was further validated in an SLE mouse model. We demonstrated that diminished PARN levels during SLE development promote the usage of distal poly(A) sites in *Foxp1* transcripts and upregulate the protein expression of FOXP1 through APA. It further disrupts class switch recombination (CSR) and curbs the overproduction of high‐affinity autoantibodies, thereby mitigating tissue damage. It is worthwhile to point out that the present study cannot completely exclude the potential contribution of PARN in non‐B cells to SLE pathology. Nevertheless, based on the specificity of the AID‐Cre model and the single‐cell expression analyses, B cells appear to be the primary effector cell type in which PARN deficiency leads to class switch recombination (CSR) defects and auto‐antibody production. Future studies using lineage‐specific knockout mice (e.g., CD4‐Cre for T cells, Lyz2‐Cre for macrophages) in combination with SLE‐induced models (e.g., pristane or chronic graft‐versus‐host disease) will be necessary to directly evaluate the function of PARN in non‐B cells.

In our study, mass spectrometry identified PTBP1 and PABP1 as binding partners of PARN in B cells. PTBP1, a member of the polypyrimidine tract‐binding protein family, recognizes Py‐rich motifs at 3′ splice sites and contributes to the selection of alternative terminal exons, thereby promoting APA at these exons [53]. Recently, it was reported that PARN interacts with PTBP1 to maintain glucose homeostasis in pancreatic β‐cells [54]. Studies have shown that PARN recognizes and binds the m^7^GpppG cap via a conserved tryptophan residue within its RRM domain (Trp475 in human; Trp468 in mouse) [55, 56], while the R3H domain contributes to its structural stabilization [57]. The CAF1 domain harbors the catalytic nuclease site [58], and the CDP domain contains an arginine‐rich nuclear localization signal [59]. To determine which domains mediate interaction with PTBP1, we generated PARN deletion mutants (ΔCAF1, ΔR3H, ΔRRM, and ΔCDP). Co‐immunoprecipitation revealed that both the CAF1 and CDP domains are required for PTBP1 binding. Consistently, AlphaFold3‐based structural prediction highlighted these regions as potential PTBP1‐interaction interfaces. Due to the predicted binding sequences in PARN are highly conserved across species, it implies that the interaction is evolutionarily preserved and functionally important. Moreover, PABP1 (PABPC1) is in the poly(A)‐binding protein (PABP) family, both of which have been proven to modulate APA [60]. One study showed that PABP1 interacted with IgH mRNA and regulated the transition from mIg to sIg in plasma cells, thereby promoting the secretion of antibodies [61]. We demonstrated that PARN cooperates with PTBP1 and PABP1 to synergistically promote CSR, as knockdown of any one of these factors markedly reduced the proportion of class‐switched B cells. Further research focusing on single‐nucleotide–resolution mapping of this interface will further clarify its mechanistic role.

## 4 Conclusion

Our work links the dysfunction of PARN to both autoimmune and immunodeficiency contexts, bridging molecular mechanisms to immune disease etiology. In summary, our data implicate that PARN acts post‐transcriptionally to enforce the phenotype of SLE and could serve as a biomarker for SLE diagnosis. It is worthwhile for future studies to generalize the mechanisms in other autoimmune disorders.

## 5 Materials and Methods

### 5.1 Animals and Diets

C57BL/6N and ICR mice were obtained from Beijing Vital River Laboratory Animal Technology Co., Ltd. *PARN* ^fl/+^ mice, meticulously cultivated within the distinguished laboratory of Professor Liu at China Agricultural University. *AID* ^Cre/+^ mice were acquired from The Jackson Laboratory. The generation of PARN conditional knockout (cKO) mice involved a precise breeding scheme, crossing *AID* ^Cre/+^ mice with *PARN* ^fl/fl^ mice derived from parental *PARN* ^fl/+^ individuals. The primer sequences utilized for PCR to determine the genotypes of *PARN* ^fl/+^ and *AID* ^Cre/+^ mice are delineated in Table S1. SPF male C57 mice (7, 8 weeks old), were purchased from Vital River Laboratory Animal Technology Co., Ltd., Beijing, China. Throughout the experimental period, these mice were provided with ad libitum access to sterilized water and standard rodent chow produced from the Beijing Vital River Laboratory Animal Technology Co., Ltd., Beijing, China. All mice were meticulously bred and housed under stringent specific pathogen‐free conditions, with a controlled temperature (22°C ± 1°C) and a consistent 12‐hour light‐dark cycle within the animal facilities of China Agricultural University. The conduct of all experiments strictly adhered to the guidelines and received the requisite approval from the Institutional Animal Care and Use Committee of China Agricultural University (Approval No. Aw10112202‐5‐1). To induce the SLE mouse model, each mouse received a single intraperitoneal injection of 0.5 mL pristane (Macklin, Cat# P815856). All mice were euthanized over 3 months post‐injection for analysis.

### 5.2 Human Samples

The SLE patients, and healthy donors included for genetic analysis all provided written informed consent, and the study protocol was approved by the Ethics Committee of the Chinese People′s Liberation Army (PLA) General Hospital. All procedures were conducted in strict accordance with the Declaration of Helsinki. Peripheral blood samples were collected from these individuals and used for RT‐qPCR analysis and Sanger sequencing.

### 5.3 Cell Culture

CH12F3 cells and splenic B cells were cultured in RPMI 1640 medium (catalog number sh30809.01, Hyclone), supplemented with 5% fetal bovine serum (catalog number P30‐3302, PAN), *L*‐Glutamine (catalog number 25030081, Thermo), Sodium Pyruvate Solution (catalog number 11360070, Thermo), and Penicillin–Streptomycin Solution (catalog number 15070063, Thermo). Cell maintenance was conducted in a humidified incubator at 37°C with 5% CO_2_.

### 5.4 PCR and qPCR

DNA was extracted using the Mouse Direct PCR Kit (B40015 Selleck). A 2% agarose gel (BY‐R0100, Biowest Agarose) supplemented with 4S Red Plus Nucleic Acid Stain (A606695‐0500, BBI) was prepared, and electrophoresed samples were subsequently visualized under the UV channel using a Tanon 5200 chemiluminescence imaging system.

Total RNA extraction was achieved using TRIzol reagent (15596018CN, Invitrogen), followed by quantification of concentration using a Nanodrop spectrophotometer (Thermo Scientific). Subsequently, cDNA synthesis was conducted according to the instructions provided with the ChamQ Universal SYBR qPCR Master Mix (Q711, Vazyme). The expression levels of target gene transcripts were evaluated using a QuantStudio 5 Real‐Time PCR instrument with a 384‐well block (Applied Biosystems). AS analyses were performed utilizing a PCR amplification instrument (Eppendorf), with primers synthesized by Shanghai Sangon Biotech (as listed in Table S1). The expression level of *Gapdh* served as the control, with its value standardized to 1. Relative transcript expression levels of other samples were determined by comparing them to the control results.

### 5.5 Plasmid Construction and Transfection

The pcDNA3.0‐PARN plasmid was designed to contain the CMV promoter upstream of the PARN coding sequence, followed by a bovine growth hormone polyadenylation signal. The plasmid backbone also included an ampicillin resistance gene for bacterial selection. The PARN coding sequence was PCR‐amplified from a template plasmid using forward and reverse primers containing restriction enzyme sites compatible with the cloning strategy. The pcDNA3.0‐PARN vector backbone was linearized by restriction enzyme digestion with EcoR I and Kpn I enzymes to create compatible ends for ligation with the PCR‐amplified EGFP fragment. The linearized pcDNA3.0‐PARN vector and PCR‐amplified PARN fragment were ligated together using T4 DNA ligase. PARN truncates plasmids, the psiCHECK2 plasmid containing *Foxp1* 3′ UTR sequences, and the PARN pET28a plasmid were purchased from WZ Biosciences Inc.

The ligated plasmid was introduced into competent *E. coli* cells via heat‐shock transformation. Following transformation, the cells were plated on LB agar supplemented with ampicillin to enable the selection of positive clones. Plasmid DNA was subsequently isolated using a commercial plasmid extraction kit (Catalog#AG21032, Accurate Biology) in accordance with the manufacturer′s protocol.

For co‐transfection, a total of 2 µg plasmids was added per well, consisting of 1 µg of each plasmid. The transfection reagent GeneTwin (Biomed) was mixed with the plasmids at a ratio of 1:3 to 1:5 (plasmid: reagent, v/v). The mixture was incubated for 10 min at room temperature to allow complex formation, then slowly added dropwise to the cell culture medium. The culture medium was replaced with fresh medium 24 h post‐transfection. Cells were harvested for subsequent analysis 48 h after transfection.

### 5.6 RNA‐Seq

Total RNA was extracted from splenic B cells following the aforementioned protocol. Initially, mRNA was purified from total RNA using poly‐T oligo‐attached magnetic beads. Subsequently, following fragmentation, a transcriptome sequencing library was constructed, and the quality of the library was evaluated using an Agilent Bioanalyzer 2100 system. Index‐coded samples were clustered utilizing a TruSeq PE Cluster kit v3‐cBot‐HS (Illumina) on a cBot Cluster Generation System, adhering strictly to the manufacturer′s guidelines. Following cluster generation, the library preparations underwent sequencing on the Illumina NovaSeq platform, yielding 150 bp paired‐end reads. Post quality control procedures ensured that downstream analyses were conducted exclusively on clean, high‐quality data. Then, a reference genome index was established, and paired‐end clean reads were aligned to this reference genome utilizing HISAT2 software (version 2.0.5). FeatureCounts (version 1.5.0) was employed to quantify reads mapped to each gene. Next, the FPKM value of each gene was computed, considering both the length of the gene and the read count mapped to it. Differential expression analyses of cKO/Ctrl samples (comprising three biological replicates per condition) were executed employing the DESeq2 R package (version 1.20.0). Genes exhibiting a *P_adj_*‐value ≤ 0.05 as identified by DESeq2 were classified as differentially expressed.

### 5.7 CLIP‐Seq

Cross‐linking and immunoprecipitation (CLIP‐seq) analysis was undertaken to explore interactions between RNA and proteins. Initially, cells which were previously cultured for 3 days with or without LPS stimulation (5 µg/mL) were washed three times in phosphate‐buffered saline (PBS) supplemented with 2% bovine serum albumin (BSA). Next, cells were exposed to ultraviolet light for cross‐linking between RNA molecules and their associated proteins. Subsequent to cross‐linking, cellular lysis was performed, followed by immunoprecipitation of RNA–protein complexes using antibodies specifically targeting the protein of interest. Immunoprecipitated complexes underwent rigorous washing to eliminate non‐specifically bound RNA molecules. RNA fragments were then released from the protein–RNA complexes and prepared for high‐throughput sequencing library construction using the NEBNext Ultra II RNA Library Prep Kit. Quantification of sequencing libraries was conducted using Qubit fluorometric quantification, with quality assessment performed using the Agilent Bioanalyzer. Following quantification, libraries were subjected to sequencing on an Illumina NextSeq 500 platform to generate either single‐end or paired‐end reads. Raw sequencing data were processed to eliminate adaptor sequences and low‐quality reads through the utilization of Cutadapt and Trimmomatic software, respectively. Subsequently, the processed reads were aligned to the reference genome using the STAR aligner, with only uniquely mapped reads retained for subsequent analysis. Peak calling was carried out using CLIPper software to identify regions exhibiting enriched RNA binding.

### 5.8 ISO‐Seq

ISO‐seq was performed by Guangzhou Epibiotek Co., Ltd using the splenic B cells stimulated by LPS for 3 days. The library is obtained by connecting the sequencing adapter to the amplified cDNAs. The concentrations were loaded onto the R10.4.1 sequencing chip and sequenced on the PromethION sequencer (Oxford Nanopore Technologies, Oxford, UK) for 48 h.

### 5.9 CRIC‐Seq

Splenic B cells were isolated from wild‐type mice using the EasyStep mouse B cell isolation kit (Catalog # 19854, Stem cell). The cells were then stimulated by LPS at 5 µg/mL. After 3 days stimulation, cells were collected for CRIC‐seq library construction. The procedure was adopted as previously described [62].

### 5.10 The Electrophoretic Mobility Shift Assay (EMSA)

For binding assays, increasing amounts of PARN proteins were incubated in binding buffer (10 mm Tris‐HCl, pH 8, 25 mm NaCl, 0.1 mm EDTA, 100 ng/µL tRNA, 50 ng/µL Heparin) at 30°C for 20 min. Then the 0.2 pmol ssRNA with 5′‐Fam modification was mixed with the incubated proteins and incubated at 30°C for 15 min. The samples were then fractionated on a 6% TBE gel. Degradation assays were performed according to Niklas et al. [35]. Briefly, a concentration gradient of PARN was incubated together with 10 nm ssRNAs for 10 min at 30°C. The reactions were stopped by adding 20 µL stop solution, and the concentrations were fractionated on a 25% TBE gel. All the gels were visualized at the FAM channel using Typhoon 7000.

### 5.11 Fluorescence In Situ Hybridization (FISH)

Cells were pre‐fixed with a 3:1 methanol: acetic acid solution in PBS for 10 min at room temperature. After removal of the pre‐fixation solution, samples were fixed again with fresh fixative for 10 min. Permeabilization was performed using ice‐cold PBS containing 0.5% Triton X‐100 and 0.5% RNase inhibitor for 20 min on ice. Slides were then washed twice for 5 min each with PBS containing 0.4% Tween 20 on a rocking platform. Blocking was carried out with filtered PBS‐T containing 1% BSA (supplemented with 0.5% RNase inhibitor if performing concurrent RNA‐FISH) for 30 min at room temperature in a humidified chamber. Primary antibody incubation was performed for 4 h at room temperature. Slides were washed three times for 5 min each with PBS‐T in a 6‐well plate, followed by incubation with secondary antibody for 2 h. After one wash with 2× SSC for 5 min, hybridization was performed using a probe mixture containing 4× SSC, 20% dextran sulfate, Yeast tRNA, salmon sperm DNA, and target probe. The mixture was denatured at 80°C for 5 min, cooled, and supplemented with 15 µL formamide, 1 µL RNase inhibitor, and 0.3 µL 5% BSA. Hybridization was carried out overnight at 37°C in a dark, humid chamber. Nuclei were stained with DAPI for 10 min at room temperature in the dark.

### 5.12 Western Blotting

Total protein was extracted using cell lysis buffer (P0013, Beyotime), supplemented with PMSF (1:100, ST506, Beyotime). The protein concentration was assessed using the BCA Protein Assay Kit (23227, ThermoFisher). Subsequently, the protein lysates underwent sodium dodecyl sulfate‐polyacrylamide gel electrophoresis and were then transferred to polyvinylidene fluoride membranes (IPVH00010, Millipore). After the transfer step, the membranes were blocked with 5% BSA for 2 h, and then were incubated overnight at 4°C with primary antibodies (see Table S2). Subsequent to primary antibody incubation, the membranes were placed at room temperature for half an hour and then were washed three times with TBSt buffer. Subsequently, the membranes were exposed to secondary antibodies (see Table S2) at room temperature for 2 h. Protein visualization was achieved using a Tanon 5200 chemiluminescence imaging system following incubation with ECL Plus (PE0010, Solarbio).

### 5.13 Co‐IP

Total protein was extracted utilizing cell lysis buffer (P0013, Beyotime), supplemented with PMSF (1:100, ST506, Beyotime). Cell lysis was placed on ice for 20 min with gentle pipetting. Subsequently, the lysate underwent pre‐clearing with 10 µL of protein A/G beads (80104G, Invitrogen) at 4°C for 1 h. After pre‐clearing, 5 µg of an anti‐PARN antibody (ab183333, Abcam), an anti‐flag antibody(F1804, Sigma), an anti‐HA Rabbit antibody (H9658, Sigma), or a normal mouse IgG (2729S, CST) was added to the lysate, and the mixture was incubated overnight at 4°C. The following day, 50 µL of protein A/G beads was introduced to the lysate, which was then incubated at 4°C for 4 h. The mixture with antibodies and target proteins was subjected to five washes of 5 min each in the magnetic rack. The enriched proteins were eluted with LDS buffer (NP0007, Invitrogen) for 15 min at 70°C by vortexing at 1000 rpm with a Thermo Mixer C. The concentrations were then conducted following the aforementioned western blotting protocol. Mass spectrometry analysis was performed as previously described [15].

### 5.14 Flow Cytometry

Flow cytometry analysis was performed using a BD LSRFortessa (BD Bioscience, USA) flow cytometer equipped with three lasers (405, 488, and 640 nm) and 12 detection channels. Prior to analysis, cells were resuspended in phosphate‐buffered saline (PBS) supplemented with 2% bovine serum albumin (BSA).

For immunophenotyping experiments, cells were stained with a panel of fluorescently labeled antibodies targeting specific cell surface markers (Table S2). Briefly, cells were aliquoted into 5 mL polystyrene tubes and incubated with the appropriate antibody cocktail for 30 min at 4°C in the dark. Following incubation, cells were washed twice with PBS and resuspended in 300 µL of PBS for analysis.

For cell proliferation assays, 1 × 10^7^ cells were labeled with 2.5 µm carboxyfluorescein succinimidyl ester (CFSE, Cat#423801, Biolegend) in 2 mL of PBS staining buffer (supplemented with 0.1% BSA) for 10 min at 37°C in the dark [63]. The reaction was quenched by adding a five‐fold volume of RPMI‐1640 medium supplemented with 10% FBS. After three washes, the cells were resuspended in complete RPMI‐1640 medium and cultured for 3 days prior to flow cytometric analysis.

Data acquisition was performed using BD FACSDiva software version 8.0.1, and data analysis was conducted using FlowJo software version 10.7.1. Gating strategies were applied to exclude debris, aggregates, and dead cells based on forward scatter (FSC), side scatter (SSC), and PI staining. Fluorescence compensation was performed using single‐stained controls for each fluorochrome.

### 5.15 Treatment With RNA Polymerase Inhibitors for mRNA Stability Assay

To assess the decay rate of *Foxp1* transcript in LPS‐stimulated B cells, 1 × 10^7^ cells from each group were treated with 10 µg/mL actinomycin D. At 2 h intervals, 1 × 10^6^ cells were collected for RNA extraction and cDNA synthesis. Untreated 1 × 10^6^ of splenic B cells from the Ctrl group served as the 0‐hour control, whose transcript expression is normalized to 1.

### 5.16 Statistical Analyses

Pearson′s correlation coefficients (*R*) were computed based on the scores of the two samples for Mass Spectrometry (MS). Statistical analyses were performed using GraphPad Prism software (version 9.0.0), with the mean values depicted alongside error bars representing the standard deviation (SD) unless otherwise specified. Student′s *t*‐test was employed to discern significant differences between the two groups. Levels of statistical significance were denoted as follows: *P < 0.05; **P < 0.01; ***P < 0.001; ****P < 0.0001. The exclusion criterion of human RNA‐seq data was based on the PCoA analysis to minimize biological variability.

## Funding

This work was funded by the National Natural Science Foundation (Grant No. 32470606) to Juan Chen, National Natural Science Foundation (Grant No. 82502815) to Naijing Hu, Henan Province Natural Science Foundation (Grant No. 252300421131) to Juan Chen, Chinese Universities Scientific Fund (Grant No. 2025TC018) to Juan Chen, the 2115 Talent Development Program of China Agricultural University to Juan Chen, the 111 project from the Education Ministry of China (No. B18053) to Fazheng Ren, and Beijing Life Science Academy (Grant No. 2024400CA0030) to Fazheng Ren.

## Conflicts of Interest

The authors declare no conflicts of interest.

## Supporting information




**Supporting File**: advs75609‐sup‐0001‐SuppMat.docx.

## Data Availability

RNA‐seq, CLIP‐seq, ISO‐seq, CRIC‐seq, and 2p‐seq data have been deposited at GSE312680, GSE313463, GSE313542, GSE313464, and GSE313461, respectively.
