# Supplementary material for: The RNA‐Binding Protein PARN Remodeled 3′ UTR Structure Defines Poly(A)‐Loading Sites to Mediate Immunoglobulin Homeostasis
Source: Adv Sci (Weinh). 2026 May 12;13(43):e75609. doi: 10.1002/advs.75609 (PMC13336127; doi:10.1002/advs.75609)
Supplement: Supplementary file 1 — Supporting File: advs75609‐sup‐0001‐SuppMat.docx. [file ADVS-13-e75609-s001.docx]

Supporting Information


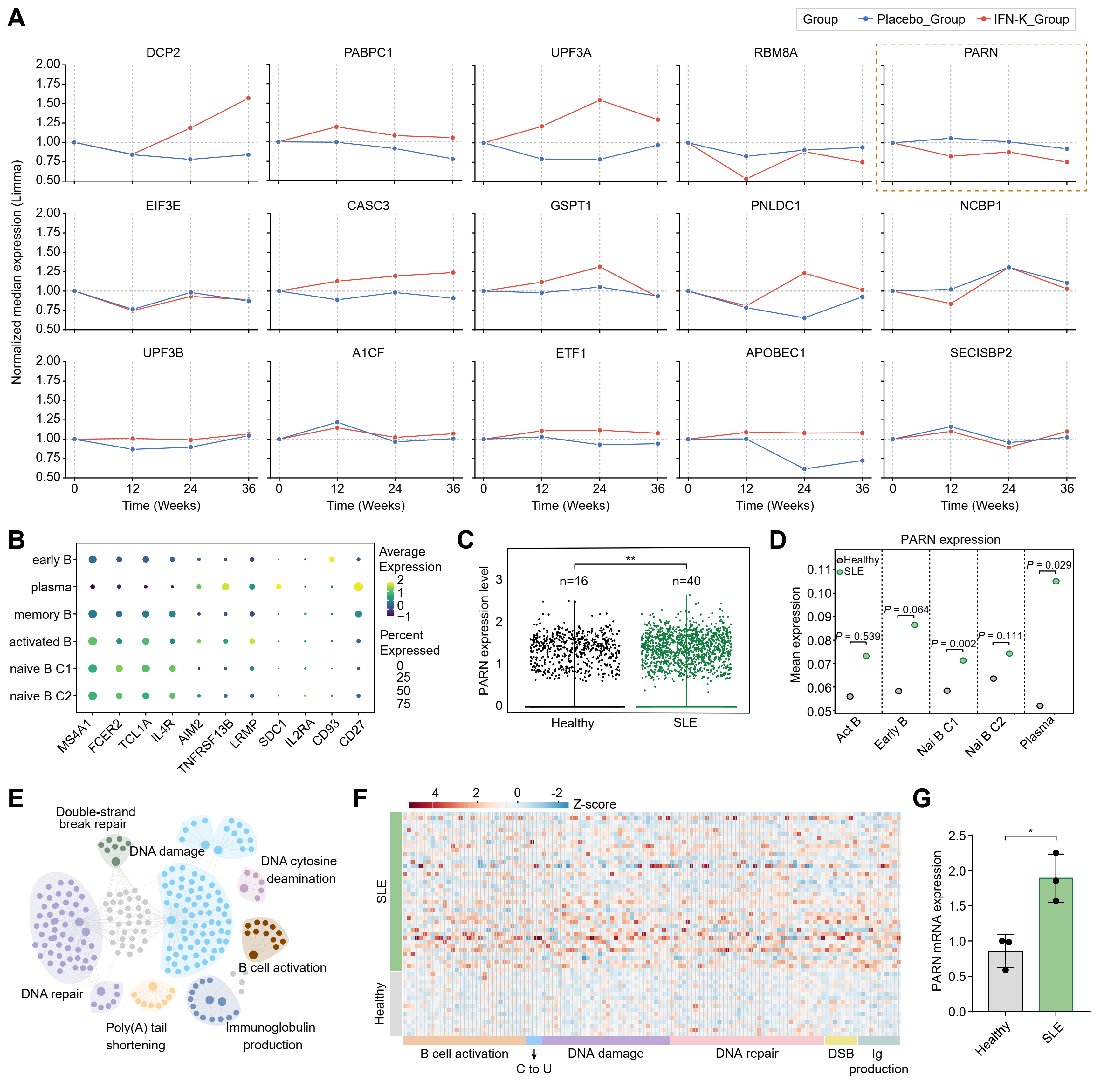


**Figure S1. Identification of PARN as a highly responsible protein in SLE patients, related to Figure 1.** (**A**) Temporal dynamics of RNA-binding protein gene expression following IFN-K treatment, derived from the GEO dataset (GSE185047). The *y*-axis indicates relative expression, normalized to baseline levels at week 0 (set as 1) for each gene. Expression values at weeks 12, 24, and 36 are presented as fold changes relative to week 0. Data points denote the median expression values at the indicated time points. IFN-K refers to interferon-α kinoid. The placebo group received a control treatment, whereas the IFN-K group received the experimental IFN-K vaccine. PARN expression graph is marked with a dashed rectangular box. (**B**) Dot plot representing the expression patterns of selected B cell type marker genes across distinct clusters. Dot color indicates the average expression level of each gene within a cluster, whereas dot size denotes the proportion of cells in that cluster expressing the corresponding gene. (**C**) Violin plot illustrating the expression of PARN in healthy controls and SLE patients (Wilcoxon rank-sum test, ** *P* < 0.01). (**D**) The expression of PARN in B cell subpopulations between healthy controls and SLE patients (Wilcoxon rank-sum test) displayed by bubble plot. (**E**) GO enrichment analysis networks showing the significant changed genes in total B cells between healthy controls and SLE patients. (**F**) Heatmap showcasing the most prominent differentially expressed genes between health control group and SLE patient group within CSR-related pathways. The color scale represents the average expression level of each gene. (**G**) RT-qPCR analysis of PARN mRNA expression in peripheral blood monocytes (PBMCs) between SLE patients and controls. Act B, activated B cells; Nai B, naïve B cells.


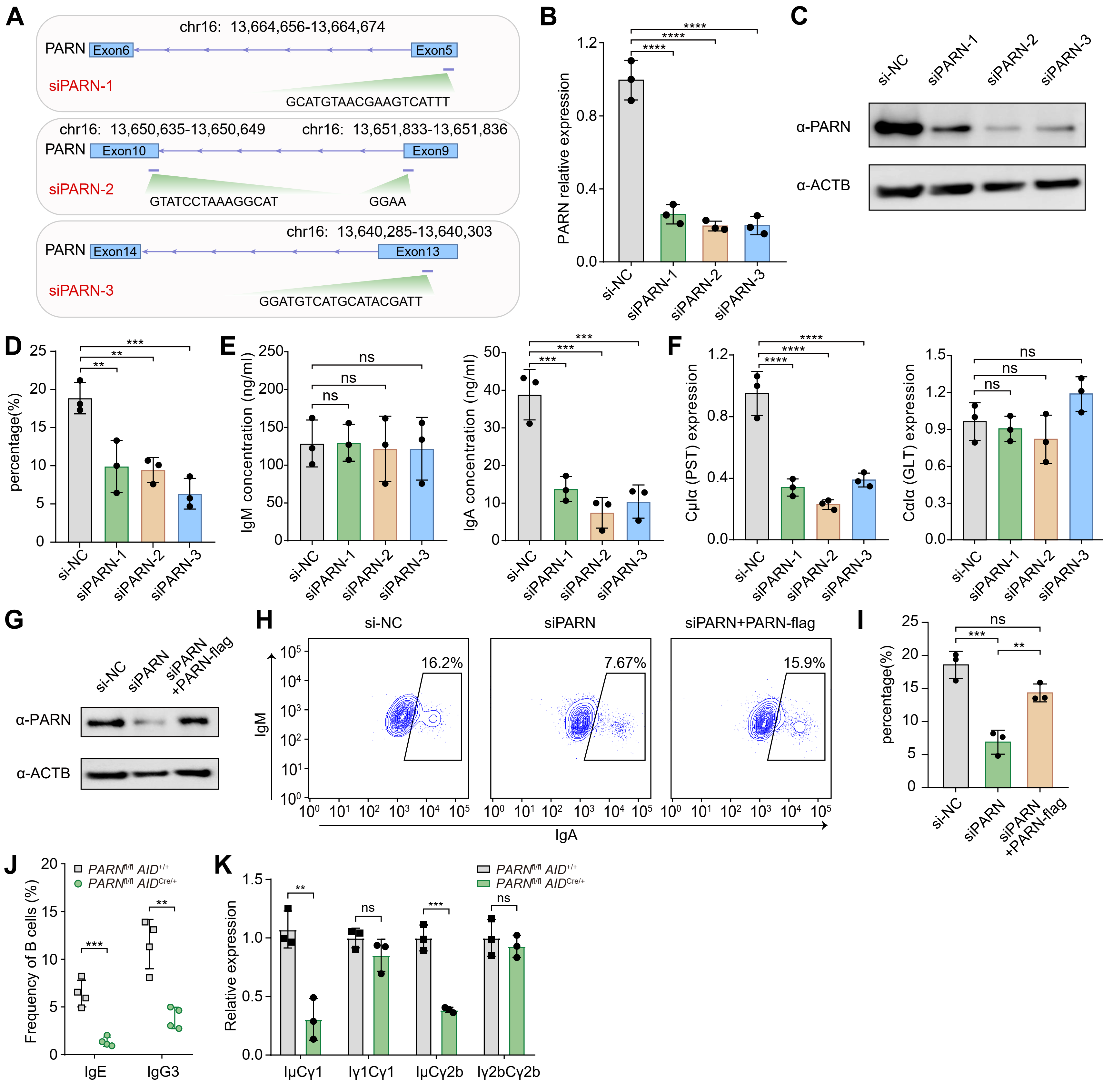


**Figure S2. Knockdown of PARN in CH12F3 cells impairs class switch recombination (CSR).** (**A**) A schematic diagram indicating the target sites of three siRNA sequences designed against PARN. (**B**) RT-qPCR analysis of PARN mRNA expression in CH12F3 cells transfected with control siRNA (NC) or the three PARN-targeting siRNAs. Expression levels were normalized to *Gapdh* transcripts and relative to the NC group (*n* = 3, mean ± SD). (**C**) Immunoblotting of PARN expression in CH12F3 cells following transfection with NC or the three PARN siRNA groups. (**D**) Proportions of IgA^+^ cells in NC and the three PARN siRNA groups after 48 hours of CIT stimulation. (*n* = 3, mean ± SD). (**E**) Concentrations of IgM and IgA in CH12F3 cell culture supernatant between NC and the three PARN siRNA groups. Each symbol represents technical duplications and columns indicate the mean. (**F**) RT-qPCR analysis of CμIα (post-switch transcript, PST) and CαIα (germline transcript, GLT) expression in the NC and three siRNA targeting groups. (G, H) Immunoblotting of PARN expression (**G**) and flow cytometric analysis for IgA^+^ cells (**H**) in NC, siRNA, and PARN-flag plasmid rescue groups. (**I**) Proportions of IgA^+^ cells shown in (H). (*n* = 3, mean ± SD). (**J**) Proportion of *PARN*^fl/fl^ *AID*^+/+^ and *PARN*^fl/fl^ *AID*^Cre/+^ IgE^+^ and IgG3^+^ B cells following 48 hours of stimulation with LPS or LPS + IL-4. (**K**) RT-qPCR analysis of different germline transcripts following 3 days of stimulation with LPS or LPS + IL-4. Relative gene expression was quantified and normalized to Gapdh transcript levels (*n* = 3). * *P* < 0.05; ** *P* < 0.01; *** *P* < 0.001; **** *P* < 0.0001; ns, no significance.


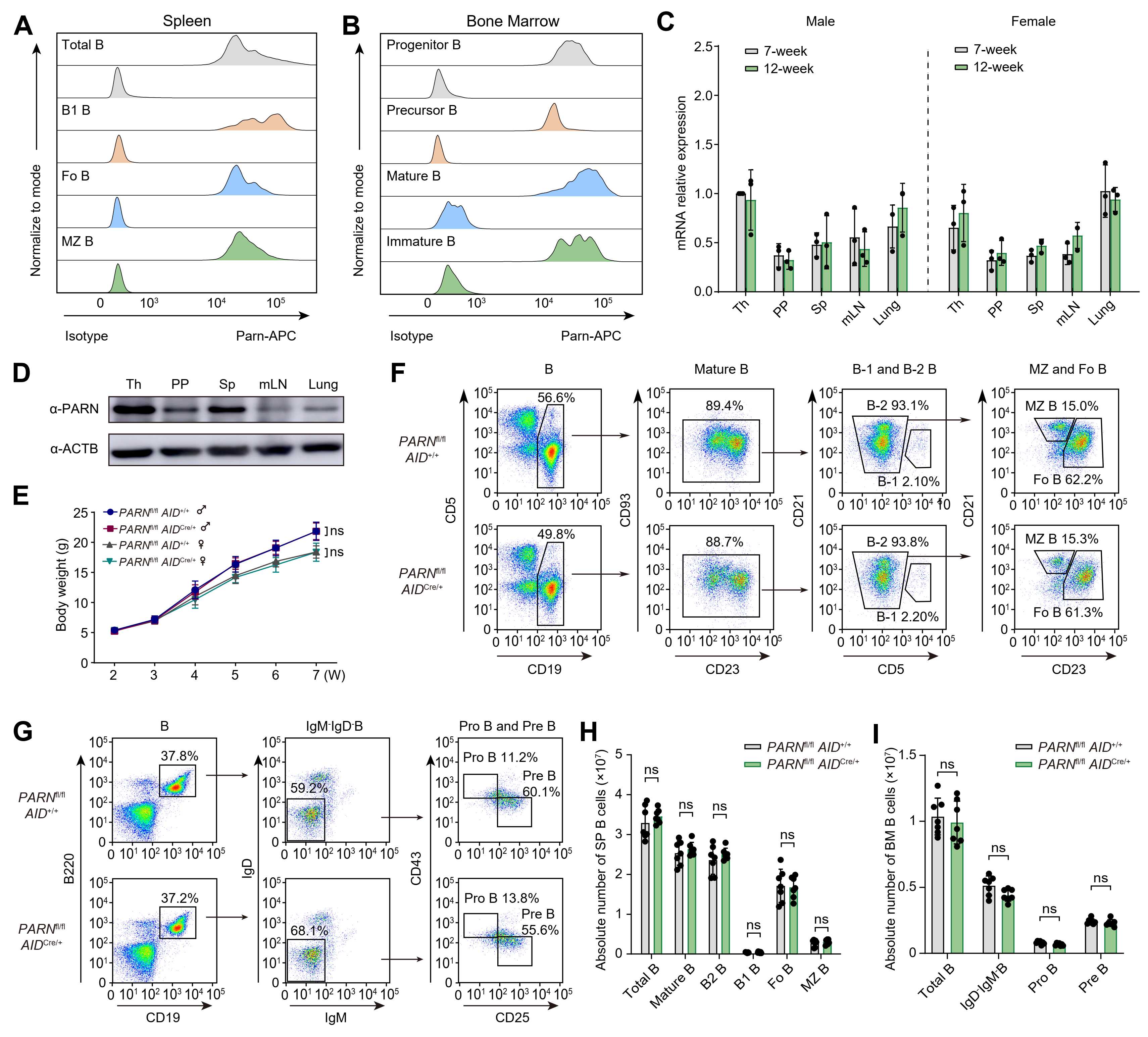


**Figure S3. Conditional knockout of PARN in activated B cells preserves normal B cell developmental processes.** (A, B) Representative histograms from intracellular flow cytometry measuring PARN in wild type B cells in spleen (**A**) and bone marrow (**B**). (**C**) Relative mRNA expression of PARN in the thymus (Th), Peyer′s patches (PP), spleen (Sp), mesenteric lymph nodes (mLN), and Lung of male and female mice at 7 and 12 weeks of age, as determined by RT-qPCR (*n* = 3 per group). (**D**) Immunoblotting of PARN protein expression in different organs of wild-type mice. (**E**) Body weight of *PARN*^fl/fl^ *AID*^Cre/+^ mice and littermate controls. Results are expressed as means ± SD (*n* = 6). Data were analyzed using a two-way ANOVA with Tukey′s multiple comparison test. (F, G) Representative pseudo-color plots from flow cytometric analysis of B cell at different stages in spleen (**F**) and bone marrow (**G**) in control and *PARN*^fl/fl^ *AID*^Cre/+^ mice. Numbers on flow cytometry pseudo-color plots indicate the percentage of gated cells. (H, I) Absolute number of control (*n* = 7) and *PARN*^fl/fl^ *AID*^Cre/+^ (*n* = 7) B cells at different stages in spleen (**H**) and bone marrow (**I**) measured by flow cytometry as shown in (F, G). Flow cytometry data for control and *PARN*^fl/fl^ *AID*^Cre/+^ samples were compared by a two-way ANOVA with Tukey′s multiple comparison test. Symbols indicate biological replicates, and bar tops represent means. ns, not significant (*P*  >  0.05).


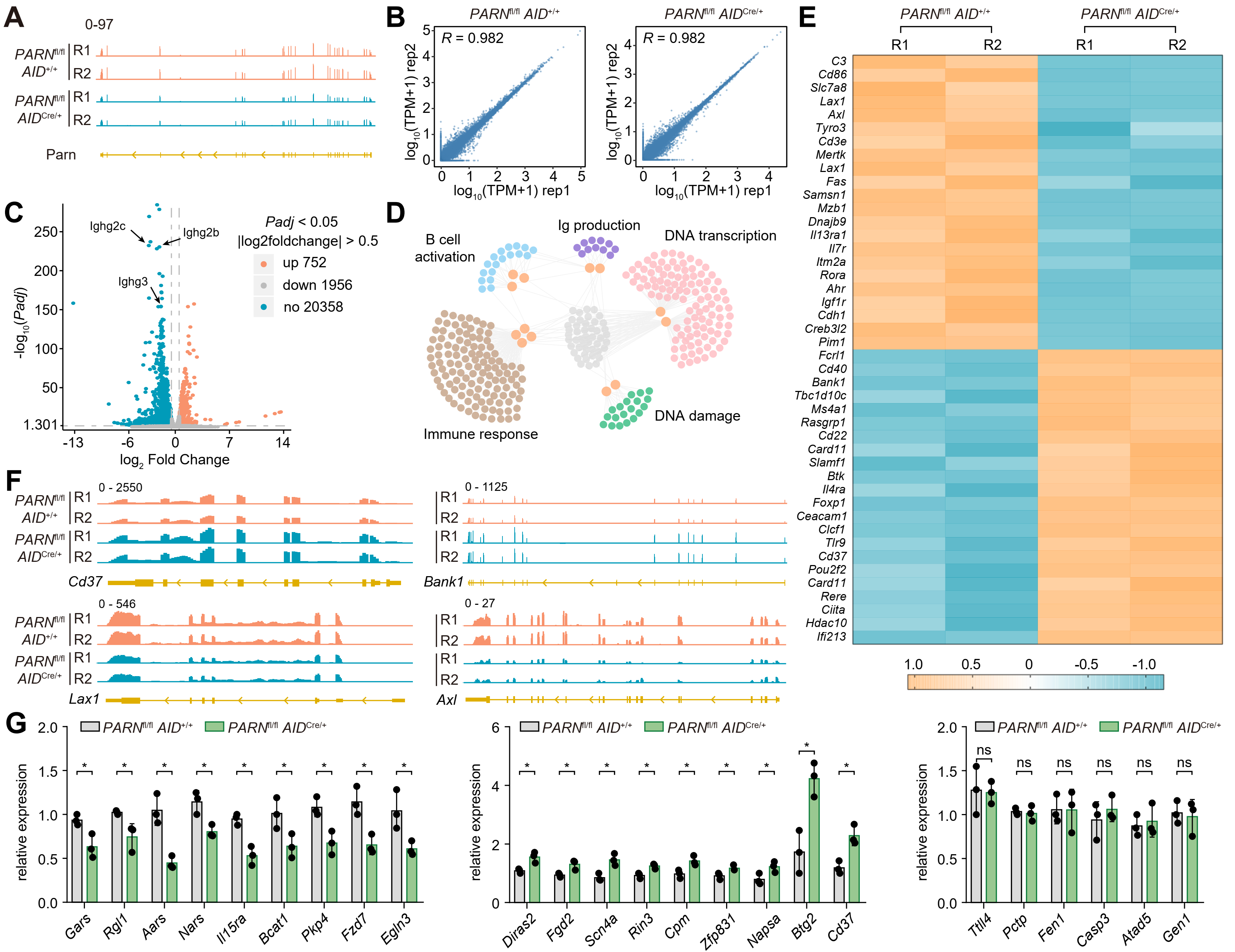


**Figure S4. PARN shapes the transcriptional landscape of CSR-related pathways in activated B cells.** (**A**) Coverage tracks showing the expression of PARN in LPS-stimulated *PARN*^fl/fl^ *AID*^+/+^ (Ctrl) and *PARN*^fl/fl^ *AID*^Cre/+^ (cKO) splenic B cells. (**B**) Scatterplot of Pearson correlation between two replicates of Ctrl and cKO samples in the RNA-seq data. (**C**) Volcano map displaying the distribution of differentially expressed genes from RNA-seq data. The abscissa in the figure represents the gene fold change in Ctrl and cKO splenic B cells after LPS stimulation. |log2 Fold Change| ≥ 0.5. *Padj* ≤ 0.05. Upregulated genes are shown as red dots, and downregulated genes are shown as green dots. (**D**) Network displaying Gene Ontology (GO) enrichment of differentially expressed genes. (**E**) Heatmap illustrating differential transcript expression within B cell activation, immunoglobulin production, and DNA damage pathways. Expression counts are row-normalized by Z score. (**F**) RNA-seq tracks represent the expression of B cell activation-associated genes which are significantly changed in the cKO group (*P* < 0.05). (**G**) RT-qPCR analysis for transcripts including downregulated (left), upregulated (middle), and not significantly changed (right) mRNAs in splenic B cells 3 days after stimulation with LPS. Relative gene expression was quantified and normalized to *Gapdh* transcript levels (*n* = 3). * *P* < 0.05; ns, no significance.


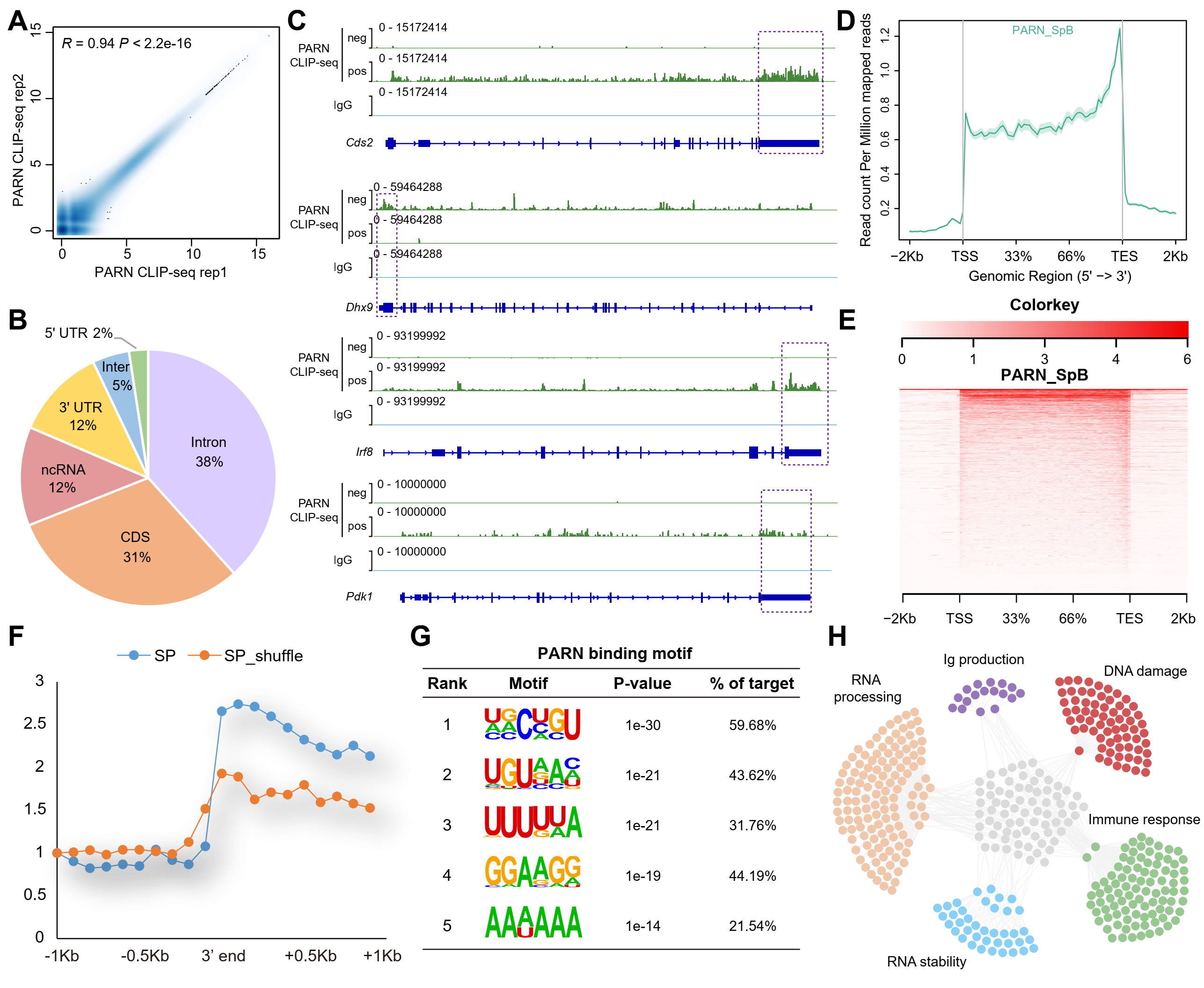


**Figure S5. The CLIP-seq profiling analysis identifies PARN-binding motifs in primary splenic B cells.** (**A**) Correlation between two replicates of primary splenic B cells. (**B**) Genomic distribution of PARN CLIP-seq peaks. (**C**) The PARN-binding peaks of transcripts. (**D**) Metagene analysis depicting the binding distribution of PARN across the genomic region spanning from the transcription start site (TSS) to the transcription end site (TES). (**F**) 3′ UTR distribution of the AATAA motif within sequencing reads. Randomly shuffle the locations of the same size of the target motif to establish control. (**G**) Consensus sequence motifs in the 3′ UTR of PARN mRNA targets, as predicted by HOMER analysis. The top five enriched motifs are shown. (**H**) Gene ontology (GO) enrichment network showing PARN binding transcripts and their related pathways.


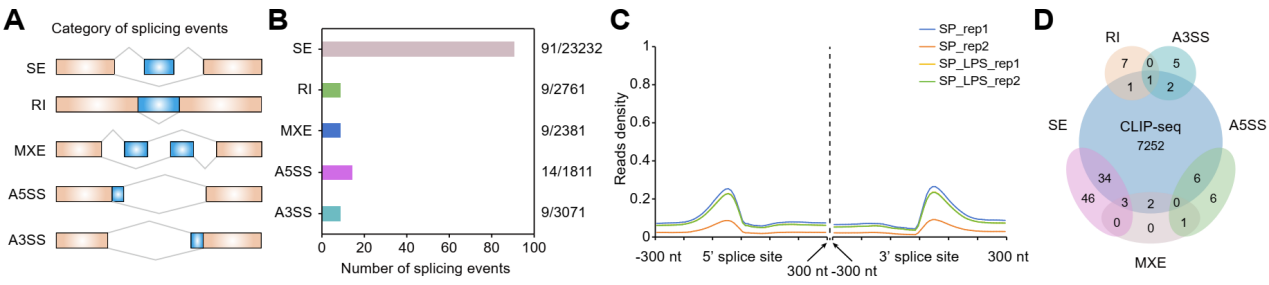


**Figure S6. Alternative splicing events are not markedly altered upon PARN depletion.** (**A**) Schematic representation of major categories of alternative splicing events. (**B**) Quantification of splicing events across canonical categories. (**C**) Metagene analysis depicting the read density of PARN binding around 5′ and 3′ splice sites in CLIP-seq data from LPS-stimulated and unstimulated splenic B cells. (**D**) Venn diagram illustrating the overlap between PARN-bound genes and differentially spliced genes across the five canonical splicing categories in *PARN*^fl/fl^ *AID*^Cre/+^ and control groups.


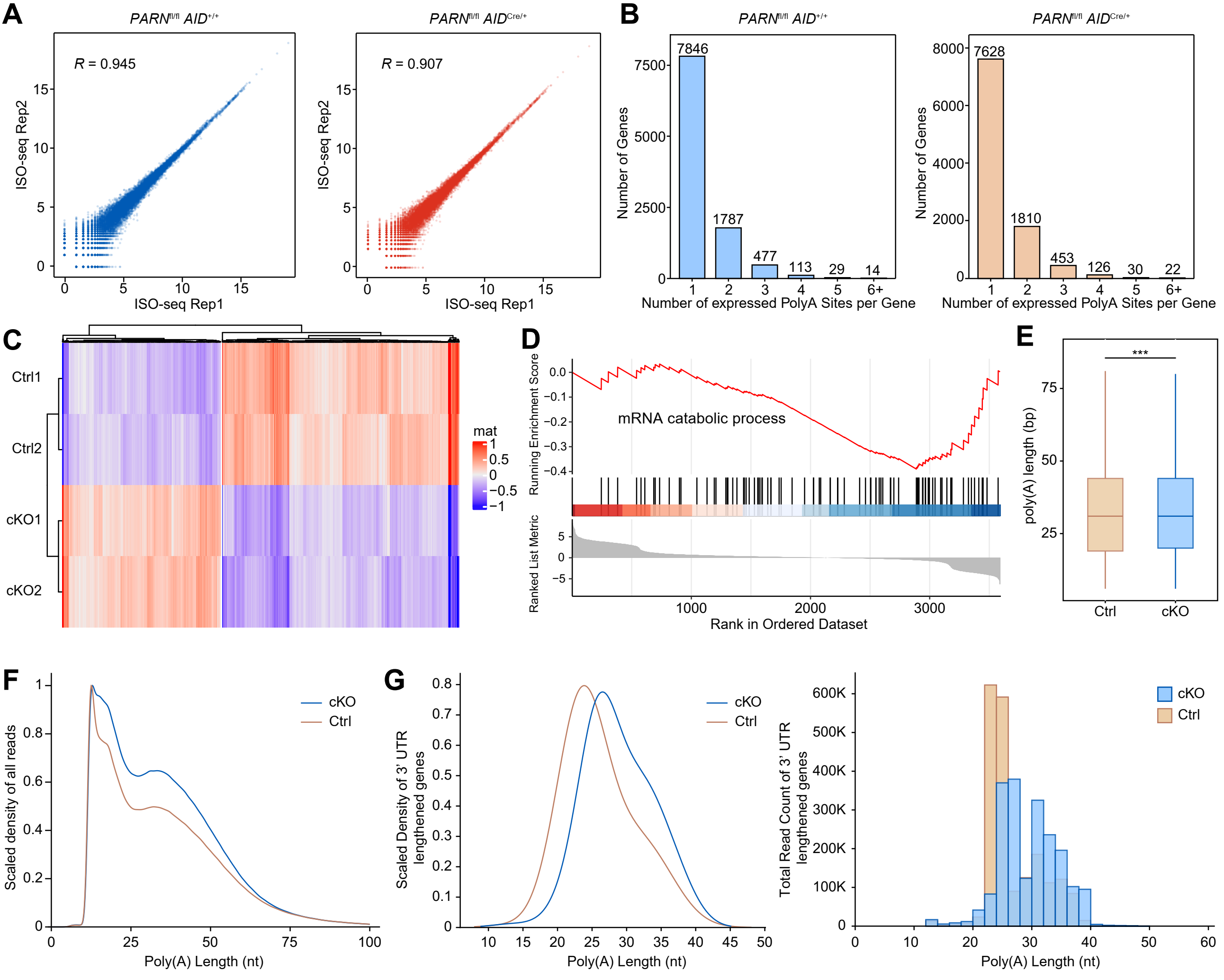


**Figure S7. 3′ UTR differentiated transcripts enriched pathways and poly(A) length profile from ISO-seq data.** (**A**) Correlation between two replicates of LPS-stimulated *PARN*^fl/fl^ *AID*^+/+^ (Ctrl) and *PARN*^fl/fl^ *AID*^Cre/+^ (cKO) splenic B cells. (**B**) Bar plots displaying the genes detected with different numbers of poly A sites. (**C**) Heatmap depicting differential transcript expression in ISO-seq data. Expression counts are row-normalized by Z score. (**D**) Gene set enrichment analysis (GSEA) for downregulated mRNA catabolic process pathway from the molecular signatures database (MSigDB). The *y*-axis represents the value of the ranking metric; the *x*-axis represents the rank for all genes. Lower levels of △CI are represented in shades of blue and higher expression is represented in red. (**E**) Comparison of poly(A) tail length across different groups. (F, G) Poly(A) length distribution for all reads (**F**) and 3′ UTR lengthened genes (**G**).


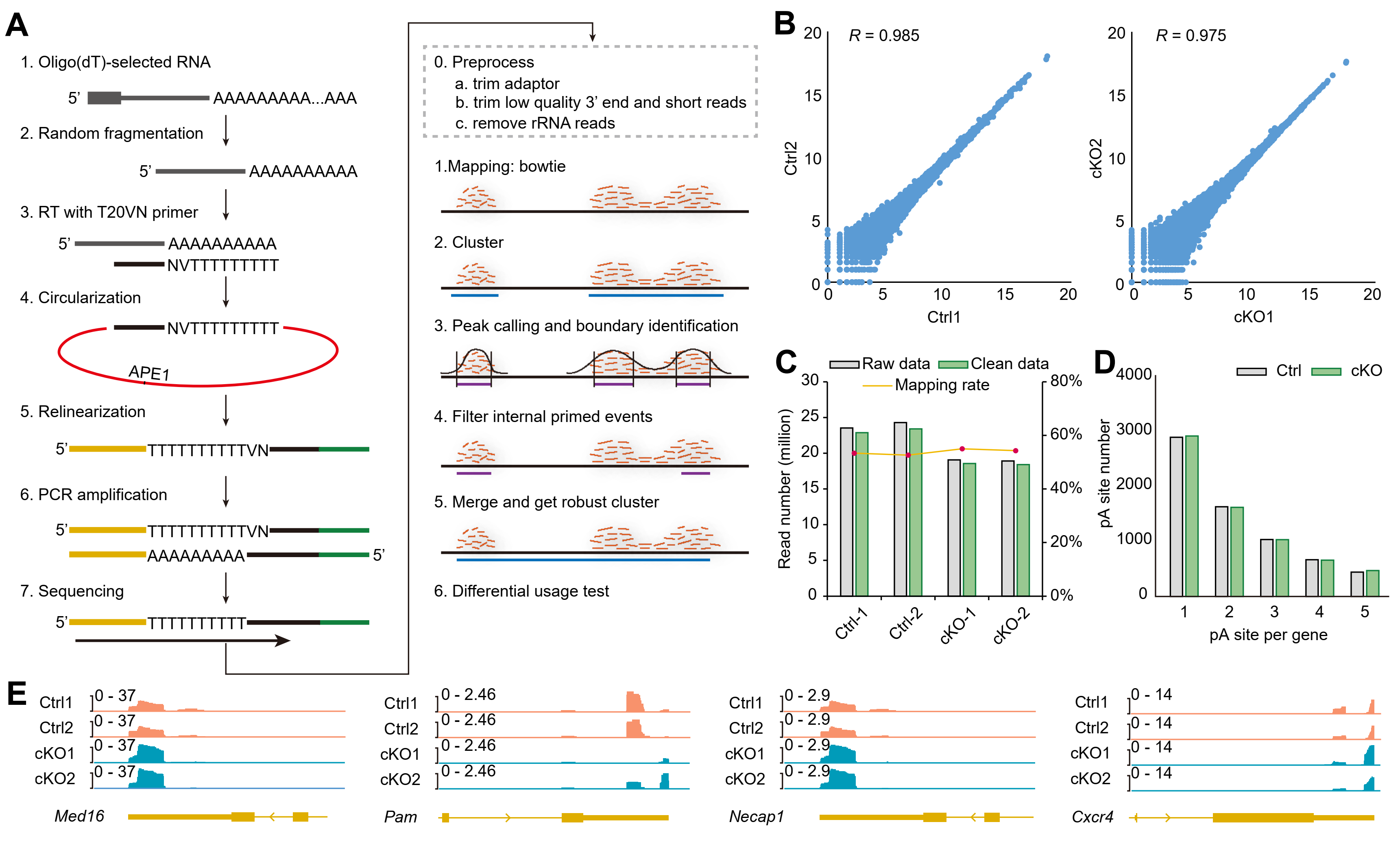


**Figure S8. 2p-seq method was performed to visualize the profile of transcripts with different lengths of 3′ UTR.** (**A**) Schematic workflow of 2p-seq. (**B**) Correlation between two replicates of LPS-stimulated *PARN*^fl/fl^ *AID*^+/+^ (Ctrl) and *PARN*^fl/fl^ *AID*^Cre/+^ (cKO) splenic B cells. (C, D) Bar plots displaying the genes with different read numbers (**C**) and poly A sites (**D**). (**E**) Coverage tracks showing the expression of different 3′ UTR isoforms in LPS-stimulated Ctrl and cKO splenic B cells.


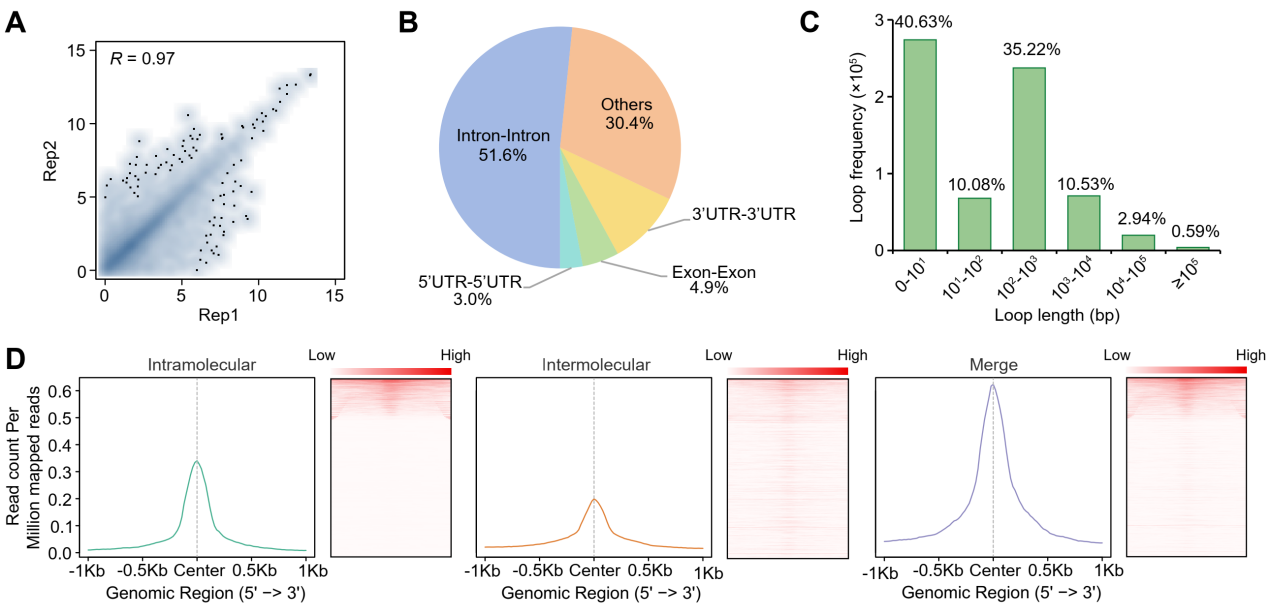


**Figure S9. RNA-RNA interactions across the transcriptomics from CRIC-seq data.** (**A**) Pearson′s correlation analysis shows a high correlation for PARN CRIC-seq between two biological replicates. Each dot represents an RNA. (**B**) RNA-RNA contact landscape for intramolecular PARN CRIC-seq chimeric reads. (**C**) The distribution of spanning distance for intramolecular RNA-RNA spatial contacts. (**D**) Metagene profile depicting the overlapping area between PARN CLIP-seq and CRIC-seq data across the whole genome. Intramolecular (left), intermolecular (middle), and merged (right) fragments within individual genes are ordered in descending gene length. Color intensity indicates the overlapping density.


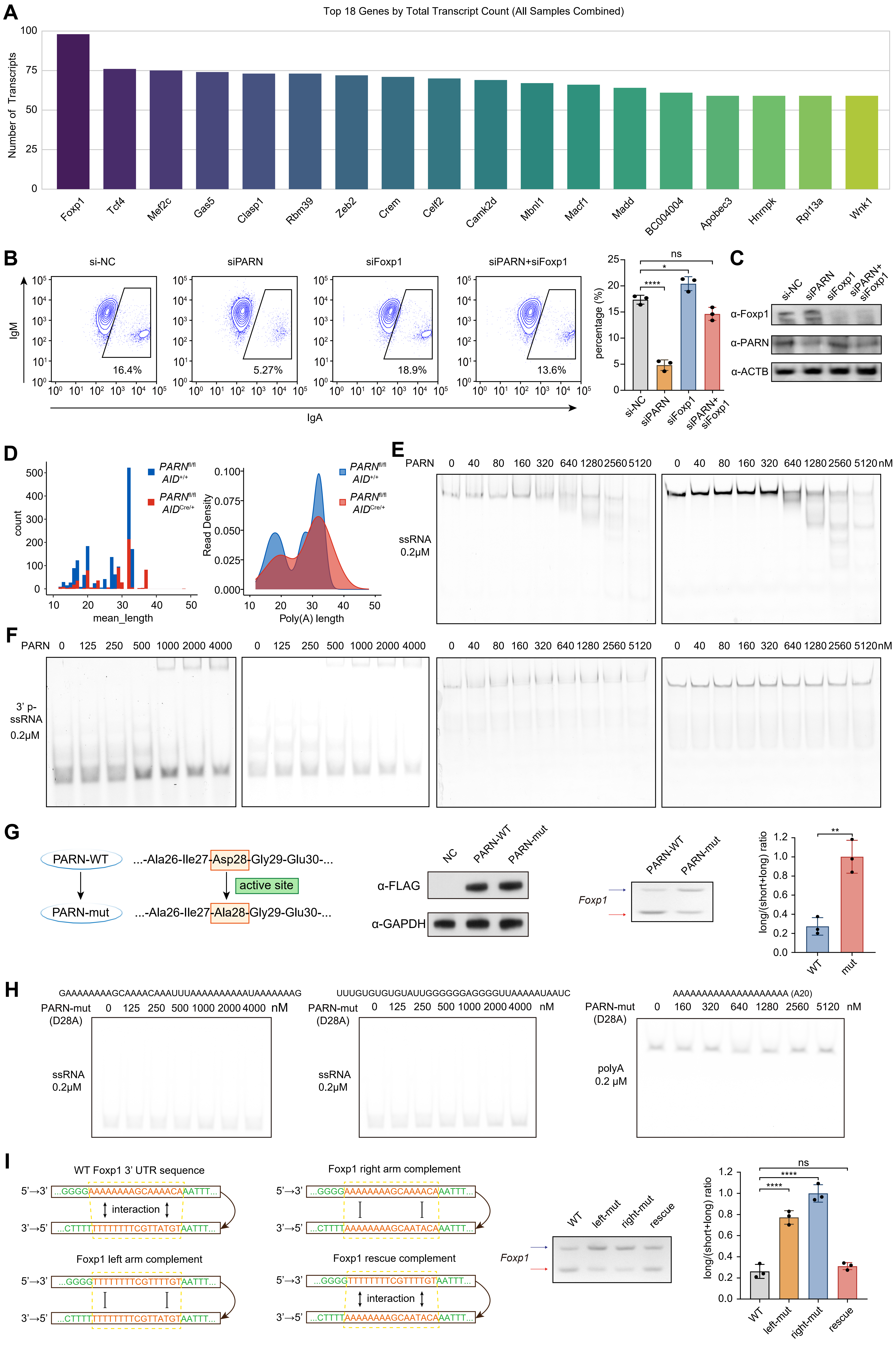


**Figure S10. *Foxp1* serves as a downstream effector of PARN through RNA-binding sites.** (**A**) Top 18 genes with high expression levels across the groups. (**B**) Flow cytometric analysis for the proportions of IgA^+^ cells in NC, siPARN, siFoxp1, and siPARN+siFoxp1 groups. (*n* = 3, mean ± SD). (**C**) Immunoblotting of PARN and Foxp1 expression from cell groups in (C). (**D**) Bar plot and curve density graph showcasing the poly(A) length distribution among different *Foxp1* transcripts in Ctrl and cKO groups. (**E**) Electrophoretic mobility shift assay showing single-stranded RNA (ssRNA) binding activities of PARN across a concentration gradient of PARN. (**F**) ssRNA binding activities and its degradation with the 3′ phosphate modification across a concentration gradient of PARN. (**G**) The alternative polyadenylation (APA) events in Foxp1 gene with or without PARN protein mutations. RT-PCR showing different isoforms of Foxp1 in 293T cells transfected by PARN and its mutant (D28A) plasmid. (student *t*-test, ** *P* < 0.01, *n* = 3) (**H**) Electrophoretic mobility shift assay showing single-stranded RNA (ssRNA) binding activities and deadenylation activity of PARN mutant across a concentration gradient of 0.2 μM ssRNA and poly(A), respectively. (**I**) RT-PCR showing different isoforms of Foxp1 in 293T cells confirming the interaction of PARN and its target 3′ UTR sequences. (one-way ANOVA, **** *P* < 0.0001, *n* = 3)


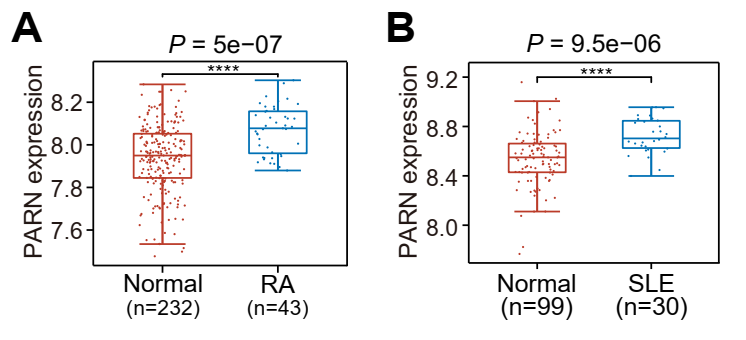


**Figure S11. Human samples reveal the correlation between PARN and diseases with imbalance of antibody production.** (**A, B**) Expression levels of PARN in two common autoimmune diseases—rheumatoid arthritis (RA) and systemic lupus erythematosus (SLE) .


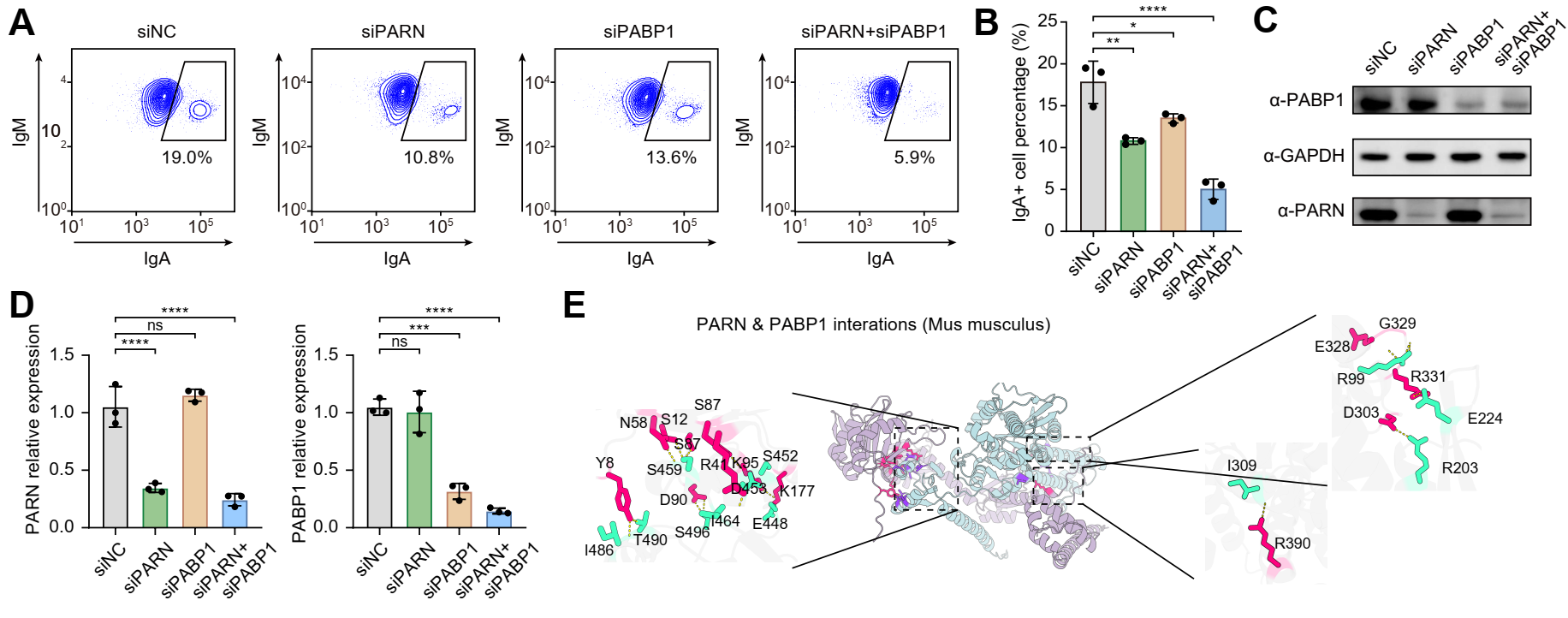


**Figure S12. Validation of PARN cofactors in CSR**. (**A**) Representative contour plots from flow cytometric analysis of IgA^+^ cells in CH12F3 cell lines following transfection with NC, siPARN, siPABP1 and siPARN+siPABP1. (**B**) Bar plots showing the proportions of IgA^+^ cells, measured by flow cytometry as shown in (A). (C, D) Immunoblotting (**C**) and RT-qPCR (**D**) analysis of PARN, PABP1 expression levels in CH12F3 cell lines following transfection with NC, siPARN, siPABP1 and siPARN+siPABP1. The expression levels were normalized to *Gapdh* transcripts and the NC group in quantitative PCR analysis (*n* = 3, mean ± SD) (**E**) Structural prediction of the interaction site of PARN and PABP1 proteins in the species of *Mus musculus* using AlphaFold 3.

**Table S1. Primers used in this study**

| PCR primer |  |  |  |  |
| --- | --- | --- | --- | --- |
| Category | Gene | Forward primer sequences(5′-3′) | Reverse primer sequences(5′-3′) |  |
| genotyping | PARN flox | CACTTGAGAGGCAGTGAATTTGAGGC | TGGATCTGGAGTTCTGGGCATCTG |  |
| genotyping | PARN flox′ | CTGATTGTGGCAGGATTTCTGTCTTTG | GAGCACATCTGTTTGTCAAAGACTTCACTG |  |
| genotyping | AID-Cre | GGACATGTTCAGGGATCGCCAGGCG | GCATAACCAGTGAAACAGCATTGCTG |  |
| APA primer |  |  |  |  |
| Category | Gene | Forward primer sequences(5′-3′) | Reverse primer sequences(5′-3′)-1 | Reverse primer sequences(5′-3′)-2 |
| APA primer | Foxp1 | CTTTGAACTGCAGCTTCGC | CAATACACACACAAAGGCCTT | ACATTCATCACAAGGTACAGAA |
| APA primer | Plek | GATGTGCCACTGGTGGCAT | CAGAACAAAGTCCTAAATCAA | CGATACAGAGAAACCCTGTC |
| APA primer | Capzb | AGACCTCTGCTTCACGCTAA | AAGATGGAGGGAGCGGTGT | GGACATCACTCCTTCCTCCA |
| APA primer | Gosr2 | TCGTGGCTTAGTATCAAACCT | GCCAGTGGCTAGAAGCAAG | GCTCATGGCTACTTTCAAAGT |
| APA primer | Pnpk | AAGCAGTTTGAGCCGCCCA | TGTTTATTGAGGGCTCAGCC | CTGAAACCTGGTGCTGGCT |
| qPCR primer |  |  |  |  |
| Category | Gene | Forward primer sequences(5′-3′) | Reverse primer sequences(5′-3′) |  |
| antibody related gene | Iγ3-Cγ3 | AACTACTGCTACCACCACCACCAG | ACCAAGGGATAGACAGATGGGG |  |
| antibody related gene | Iγ1-Cγ1 | GGCCCTTCCAGATCTTTGAG | ATGGAGTTAGTTTGGGCAGCA |  |
| antibody related gene | Iγ2b-Cγ2b | GATGGGGAGGAGTTGGCAGAT | CGGAGGAACCAGTTGTATC |  |
| antibody related gene | Iε-Cε | ACTAGAGATTCACAACG | AGCGATGAATGGAGTAGC |  |
| antibody related gene | Iα-Cα | CAAGAAGGAGAAGGTGATTCAG | GAGCTGGTGGGAGTGTCAGTG |  |
| antibody related gene | Iμ-Cγ3 | CTCGGTGGCTTTGAAGGAAC | ACCAAGGGATAGACAGATGGGG |  |
| antibody related gene | Iμ-Cγ1 | ACCTGGGAATGTATGGTTGTGGCTT | ATGGAGTTAGTTTGGGCAGCA |  |
| antibody related gene | Iμ-Cγ2b | ACCTGGGAATGTATGGTTGTGGCTT | CGGAGGAACCAGTTGTATC |  |
| antibody related gene | Iμ-Cε | CTCGGTGGCTTTGAAGGAAC | AGCGATGAATGGAGTAGC |  |
| antibody related gene | Iμ-Cα | ACCTGGGAATGTATGGTTGTGGCTT | TAATCGTGAATCAGGCAG |  |
| common gene | PARN mouse | CCAAAGAGTGGAAAACCAGCGAC | TGGCTGAGAGAAACGAAGGCTG |  |
| common gene | PARN deletion | CTCCAGCATTGACTTCCTGG | GTCAATAAACTTCTTCTGATCC |  |
| common gene | PARN human | CCTTGCGGAATTGGAAAAGCGG | CTCGTGGAGTTGTTCAGAGGCT |  |
| common gene | GAPDH mouse | CATCACTGCCACCCAGAAGACTG | ATGCCAGTGAGCTTCCCGTTCAG |  |
| common gene | GAPDH human | GTCTCCTCTGACTTCAACAGCG | ACCACCCTGTTGCTGTAGCCAA |  |
| RNA-seq validation | Diras2 | AAGTGCGCCTTCATGGAGACGT | CTCTTCTTGCCGTCGATCTGGA |  |
| RNA-seq validation | Fgd2 | TGACCGATGCTGAGTTCCCACA | AGCAGCCTTGAAGGTCTCACTC |  |
| RNA-seq validation | Scn4a | TTCTCGGAGCCTGAGGACATCA | GTGAAACACTCCTCAGGTAGCTC |  |
| RNA-seq validation | Rin3 | ACCTGCTACAGAGCACTGAACTC | TACAAGGCTGACTCCACTATGGC |  |
| RNA-seq validation | Cpm | ATGGAGGCGTTCCTAAAGAGCG | CTCTGTGTTCCTTTGGAGTCTGC |  |
| RNA-seq validation | Zfp831 | GAGGAGAAGTGTCTACACAGGC | CGAGATGTCTTCACCTTTCTACG |  |
| RNA-seq validation | Napsa | CACAGGACCTAGTGAGGAGATC | AACCAGACTCCACCAAGGTGGA |  |
| RNA-seq validation | Btg2 | GAGCGAGCAGAGACTCAAGGTT | CGATAGCCAGAACCTTTGGATGG |  |
| RNA-seq validation | Cd37 | TGGTGTTGAGGACGATCCAGAG | CTCGTTCGCTTTCAGCATCTGG |  |
| RNA-seq validation | Gars | GATCCTGGAGATTGACTGCACC | TCAACAGGTGGTCTGCTCGGAA |  |
| RNA-seq validation | Rgl1 | GCTCCTACAGTCTGCTTGCAAC | AGCGGCTTCAATCTCACACGAG |  |
| RNA-seq validation | Aars | CCAATCAGACTCCAGTGGTAGC | AGTGACCACCTCGTCCACAAAC |  |
| RNA-seq validation | Nars | CCTGAACCGTTTAGAGGACCTAG | CAGCCACTCAATAGCATCCGAG |  |
| RNA-seq validation | Il15ra | GACACCAAAGGTGACCTCACAG | CTGTCTCTGTGGTCATTGCGGT |  |
| RNA-seq validation | Bcat1 | CTGCCTCTGTTTTGCACTACGC | TCCTCACAGCAGATCGGCACAT |  |
| RNA-seq validation | Pkp4 | CTGATGACGGTGCCACAAGATC | CATTTGCCTGGACCGATGGGAA |  |
| RNA-seq validation | Fzd7 | GATGGTGACCTACTCAGTGGAG | ATGGTGCGGATGCGAAAGAGAG |  |
| RNA-seq validation | Egln3 | CAACTTCCTCCTGTCCCTCATC | CCTGGATAGCAAGCCACCATTG |  |
| RNA-seq validation | Ttll4 | CTTCGGTGGAAGATGAGCACAG | CCAGGAGACTTCATGTGGTGAC |  |
| RNA-seq validation | Pctp | TACCCTTTCCCGCTGTCCAACA | GGAAACTGAGGTGCCGAGATAC |  |
| RNA-seq validation | Fen1 | ACCAAGAGGCTCGTGAAGGTCA | GCAGCATAGACTTTGCCAGCCT |  |
| RNA-seq validation | Casp3 | GGAGTCTGACTGGAAAGCCGAA | CTTCTGGCAAGCCATCTCCTCA |  |
| RNA-seq validation | Atad5 | GACCAAAAGAGCAGCCATTCCTG | CAGTAAATCTGGCAAACCACTCAT |  |
| RNA-seq validation | Gen1 | CCCGAGTCAGAAATGGAGTCCA | CGCTTCTTCCATTGTAAGGAGGC |  |
| co-IP related gene | Ptbp1 | CACCGCTTCAAGAAACCAGGCT | GTTGCTGGAGAAGAGGCTCTTG |  |
| co-IP related gene | Pabpc1 | TGCAGAGGATGGCAAGTGTACG | GCTAGGAGGATAGTATGCAGCAC |  |
| co-IP related gene | Foxp1-Total | CTTTGAACTGCAGCTTCGC | AGGTCGTCCACTGGAGTCT |  |
| co-IP related gene | Foxp1-Long | TTCTGTACCTTGTGATGAATGT | CTTGAGAGAAACATGGTGATG |  |

**Table S2. Antibodies used in this study**

| **Category** | **Antibody** | **Company** | **Information** |
| --- | --- | --- | --- |
| Western blotting | PARN | Abcam | ab125185 |
| Western blotting | PTBP1 | Invitrogen | 32-4800 |
| Western blotting | PABP | Proteintech | 66809-1-Ig |
| Western blotting | Beta-actin | Proteintech | 66009-1-Ig |
| Western blotting | GAPDH | Proteintech | 60004-1-Ig |
| Flowcytometry | B220 | Biolegend | Catalog#103224 |
| Flowcytometry | CD19 | Biolegend | Catalog#152418 |
| Flowcytometry | IgD | Biolegend | Catalog#405711 |
| Flowcytometry | IgM | Biolegend | Catalog#406511 |
| Flowcytometry | CD43 | Biolegend | Catalog#143203 |
| Flowcytometry | CD25 | Biolegend | Catalog#113703 |
| Flowcytometry | CD5 | Biolegend | Catalog#100641 |
| Flowcytometry | CD23 | Biolegend | Catalog#101607 |
| Flowcytometry | CD93 | Biolegend | Catalog#136505 |
| Flowcytometry | IgA | Catalog#407003 | Biolegend |
| Flowcytometry | IgG2b | Catalog#406705 | Biolegend |
| Flowcytometry | IgG3 | Catalog#406803 | Biolegend |
| Flowcytometry | PE-streptavidin | Catalog#405203 | Biolegend |
| Flowcytometry | IgG1 | Catalog#406605 | Biolegend |
| Flowcytometry | IgE | Catalog#406907 | Biolegend |
| Flowcytometry | CD95 | Catalog#152606 | Biolegend |
| Flowcytometry | GL7 | Catalog#144614 | Biolegend |
| Flowcytometry | CD138 | Catalog#142509 | Biolegend |
| Flowcytometry | CD38 | Catalog#102741 | Biolegend |
